# CAMSAP2 is required for bridging fiber assembly to ensure mitotic spindle assembly and chromosome segregation in human epithelial Caco-2 cells

**DOI:** 10.1371/journal.pone.0308150

**Published:** 2025-01-09

**Authors:** Naoko Nishizawa, Riku Arai, Koki Hiranuma, Mika Toya, Masamitsu Sato

**Affiliations:** 1 Department of Life Science and Medical Bioscience, Laboratory of Cytoskeletal Logistics, Graduate School of Advanced Science and Engineering, Waseda University, Shinjuku, Tokyo, Japan; 2 Faculty of Science and Engineering, Global Center for Science and Engineering, Waseda University, Shinjuku, Tokyo, Japan; 3 Institute for Advanced Research of Biosystem Dynamics, Waseda Research Institute for Science and Engineering, Graduate School of Advanced Science and Engineering, Waseda University, Shinjuku, Tokyo, Japan; 4 Institute for Medical-Oriented Structural Biology, Waseda University, Shinjuku, Tokyo, Japan; University of Toronto, CANADA

## Abstract

In mammalian epithelial cells, cytoplasmic microtubules are mainly non-centrosomal, through the functions of the minus-end binding proteins CAMSAP2 and CAMSAP3. When cells enter mitosis, cytoplasmic microtubules are reorganized into the spindle composed of both centrosomal and non-centrosomal microtubules. The function of the CAMSAP proteins upon spindle assembly remains unknown, as these do not exhibit evident localization to spindle microtubules. Here, we demonstrate that CAMSAP2, but not CAMSAP3, is required for spindle assembly upon mitotic entry. CAMSAP2 knockout (KO) Caco-2 cells showed a delay in mitotic progression, whereas CAMSAP3 KO cells did not. The spindle in CAMSAP2 KO cells was short and displayed a reduced microtubule density, particularly around chromosomes. This indicated a loss of bridging fibers, which are known to assist alignment of sister kinetochores through interaction with kinetochore fibers. Consistent with this, live-cell imaging of CAMSAP2 KO cells captured slow elongation of the anaphase spindle and errors in chromosome segregation. Therefore, we propose that CAMSAP2 ensures efficient reorganization of cytoplasmic microtubules into the mitotic spindle through constructing bridging fibers that assist faithful segregation of sister chromatids.

## Introduction

The mitotic spindle plays an essential role in chromosome segregation. Microtubules, microtubule-associated proteins (MAPs), and a pair of centrosomes contribute to bipolar spindle assembly. Spindle microtubules are morphologically classified into three categories. First, astral microtubules that emanate outward from the centrosomes are required to determine the position and orientation of the spindle in a cell [1]. Second, kinetochore microtubules that are associated with kinetochores, a proteinaceous mega-complex formed at the centromere of the chromosome. Kinetochore microtubules are often assembled into thick bundles called kinetochore fibers (K-fibers) [2,3]. Finally, the interpolar microtubules (aka non-kinetochore microtubules) are assembled with relatively short microtubules cross-linked with each other and interdigitated at the center of the spindle to connect the poles to each other, thereby providing structural stability to the spindle [4].

Microtubules associated with centrosomes were originally defined as the major backbone of the bipolar spindle because centrosomes serve as major microtubule-organizing centers. In the last couple of decades, evidence of non-centrosomal microtubules in the spindle has accumulated from various cell biology and biochemical studies [5]. Microtubules are assembled in close vicinity of chromosomes in accordance with the molecular gradient of Ran GTPase [6], and such microtubules around chromosomes also contribute to bipolar spindle assembly in somatic and acentrosomal cells (e.g., female meiosis) [7]. Another case involving non-centrosomal microtubules is microtubule-dependent microtubule nucleation. Augmin recruits the γ-tubulin ring complex (γ-TuRC, the nucleation base for microtubules) to the lateral surface of an existing microtubule so that another microtubule bundle can be nucleated from the surface of the microtubule lattice [8–12]. Augmin often nucleates microtubules that run parallel to sister kinetochore fibers attached to sister kinetochores in a bipolar manner [13–15]. Such microtubules, called bridging fibers, efficiently transmit the outward force of the inter-kinetochore tension generated by the kinetochore fibers [14,16–18].

Both centrosomal and non-centrosomal microtubules comprise a cytoplasmic array of microtubules in interphase and non-dividing cells. The minus-end of centrosomal microtubules is capped with γ-TuRC tethered at centrosomes, whereas the minus-end of non-centrosomal microtubules in mammalian cells are often associated with CAMSAP proteins to regulate the behavior of associating microtubules [19–22]. There are three variants of CAMSAPs in mammals, all of which have a CH domain, three coiled-coil domains (CC1–3), and a CKK domain that is critical for binding to the microtubule minus end [21–23]. *In vitro* assays identified another microtubule-binding domain, called MBD, in CAMSAP2 and CAMSAP3 that binds to and stabilizes the minus ends of microtubules, whereas CAMSAP1 tracks the minus ends without affecting the dynamics of associating microtubules *in vitro* [21]. In addition to the common domains of CAMSAP1 and CAMSAP2, CAMSAP3 has a domain that promotes the binding to and stabilizing of minus ends of the microtubules [21,22,24,25].

CAMSAPs have been well studied, particularly in epithelial cells, which has characterized their *in vivo* functions [26–34]. CAMSAP3 governs the apico-basal polarity of the microtubule array and the intracellular arrangement of organelles in the epithelial cells of the small intestine and renal proximal tubules in mice [26,28]. CAMSAPs are also required in neurons for the induction of single axons and branching of dendrites [35,36].

*Drosophila* Patronin (aka Ssp4: short spindle phenotype 4) is the founding member of the CAMSAP protein family [37,38]. Unlike CAMSAP proteins, Patronin has been well studied in the context of mitotic spindle assembly. Patronin binds to the minus end of microtubules and prevents microtubule depolymerization induced by kinesin-13. Patronin knockdown (KD) causes short spindles in mitotic S2 cells. This defect was ameliorated by the simultaneous KD of kinesin-13, demonstrating that Patronin protects the minus end from depolymerization via kinesin-13 [38]. Additionally, Patronin controls spindle elongation during anaphase B in *Drosophila* syncytial embryos by antagonism with kinesin-13 [39]. Although the knowledge of *Drosophila* Patronin implies the potential roles of mammalian CAMSAPs in the spindle, only CAMSAP1 is reported to localize to the mitotic spindle; CAMSAP1 tracks the minus ends of spindle microtubules and its depletion causes a small reduction in spindle length [40], whereas almost no preceding results have been reported that imply possible mitotic functions of CAMSAP2 and CAMSAP3. This may be due to the lack of discrete localization and microtubule-binding of CAMSAP2 in HeLa cells [21,41]. Furthermore, CAMSAP2 depleted cells reportedly do not show obvious deviations in mitosis, although the data were not disclosed [21]; therefore, the details were not clear. To determine the uncharacterized mitotic functions of mammalian CAMSAPs, we focused on the mitotic behavior of CAMSAP2- and CAMSAP3- KO Caco-2 cells derived from human colon carcinoma.

## Materials and methods

### Cell culture

Caco-2 Cells (ATCC HTB-37) were cultured at 37°C in 5% CO_2_ atmosphere, in D-MEM(Dulbecco’s Modified Eagle’s Medium)/Ham’s F-12 (FUJIFILM Wako, Osaka, Japan) with 10% FBS (v/v) containing penicillin-streptomycin (FUJIFILM Wako). For transfection, cells were incubated in D-MEM/Ham’s F-12 with 10% FBS (v/v) without penicillin-streptomycin.

### Plasmids

For the localization analysis of CAMSAPs, pEGFP-*Camsap2*, pEGFP-*Camsap3* (a gift from Masatoshi Takeichi, RIKEN BDR [19,20]), 3xGFP-CAMSAP1 plasmids (a gift from Anna Akhmanova, Utrecht University [21]) were used.

Plasmid construction for CRISPR/Cas9-mediated CAMSAP2 and CAMSAP3 KO was performed as previously described, with some modifications [42]. To construct the CRISPR/Cas9 vectors, mutually complementary oligonucleotides corresponding to the sgRNA sequences were annealed and ligated into PX459 digested with BbsI. The sgRNA sequences were as follows:

sgRNA# CAMSAP2 5’-GCTGAACTATACTGTCGTGC-3’

sgRNA# CAMSAP3 5’-GTACGATTTCTCGCGGGCCA-3’

Plasmid construction for EB1-Cterm tagging was conducted as previously described, with some modifications [43]. All primers used are listed in S1 Table. To create homology arms, approximately 1000 bp of the C-terminal region of EB1 was amplified from genomic DNA by PCR and cloned into pBluescript II KS. This product was used as a template for the second inverse PCR to create a cloning site for the insertion of a tag cassette in the middle of the homology arm. The mCherry2-NeoR cassette was excised from pMK280 by BamHI digestion. The digested cassette was cloned into the inverse PCR products to construct a donor plasmid containing the mCherry2-NeoR cassette flanked by a pair of homology arm sequences corresponding to the C-terminus of the EB1 coding region. pMK280 containing the mCherry2-NeoR cassette was a gift from Masato Kanemaki (National Institute of Genetics). To construct the CRISPR/Cas9 vectors, mutually complementary oligonucleotides corresponding to the sgRNA sequence (sgRNA# EB1-Cterm 5’- GTTATCCTTAGAGGACTCAC-3’) were annealed and cloned into BbsI-digested PX330 via Gibson assembly.

### Stable cell lines

CAMSAP2 KO and CAMSAP3 KO Caco-2 cell lines were generated as previously described, with some modifications [42]. Cells were transfected with PX459 harboring sgRNAs using the Neon transfection system or Lipofectamine LTX (Invitrogen). For selection, transfected cells were incubated with 20 μg/mL puromycin (InvivoGen, California, USA) for 2 days. After puromycin removal, the cells were diluted in 10 cm dishes to isolate single colonies.

EB1-mCherry2 cell lines were generated as previously described, with some modifications [43]. The cells were transfected with the donor and CRISPR/Cas9 plasmids (see above) using the Neon transfection system. On the day following transfection, the cells were diluted in 10 cm dishes and cultured in a medium containing G418 for 13–14 days for selection. Tag insertion was confirmed by PCR and live-cell imaging.

### siRNA transfection

Cells were transfected with 5 nM siRNAs designed to specifically target the protein of interest using Lipofectamine RNAiMAX according to the manufacturer’s instructions. Silencer Select siRNA (Thermo Fisher Scientific, Waltham, Massachusetts, USA) targeting KIF2A and HAUS6 with the following sequences, as well as Negative Control No.1 siRNA (Thermo Fisher), were purchased:

si KIF2A 5’-GGAATGGCATCCTGTGAAA-3’ [44]

si HAUS6 5’-CAGUUAAGCAGGUACGAAATT-3’ [13]

### Cell synchronization and phosphatase assays

Cells were synchronized to mitosis as described previously, [21] with some modifications. Briefly, S-trityl L-cysteine (STLC, an inhibitor of mitotic kinesin Eg5) (Sigma-Aldrich, St. Louis, MO, USA) was used to synchronize cells to mitosis. Both wild type (WT) and CAMSAP2 KO Caco-2 cells were seeded into two wells of a six-well plate (IWAKI, Shizuoka, Japan) and were incubated in media containing 5 μM STLC for 16 hours. The cells were washed with ice-cold 1 mM EDTA/PBS. After removing the buffer, cells in both wells were suspended and collected using total 150 μL of ice-cold lysis buffer (20 mM HEPES at pH 7.4, 1 mM EDTA, 150 mM NaCl, 1% Triton X-100, 0.1% sodium deoxycholate, 0.1% SDS, 10 mM NaF, 1 mM Na_3_VO_4_ and a protease inhibitor cocktail) to prepare cell lysates.

Cell lysates were prepared and used for phosphatase treatment [45]. Cell lysates (approximately 150 μL/cell line) were then transferred into 1.5 mL microtubes and centrifuged at 4°C, 15000 rpm, for 10 min. After centrifugation, each supernatant was dispensed into three tubes (34 μL/tube). Subsequently, λ protein phosphatase (λPP) and the other reagents (Table 1) were added to each tube. All the samples were incubated at 30°C for 30 min. After incubation, 13 μL of 5× sample buffer was added to each sample and samples were boiled at 98°C for 8 min. Subsequently, the samples were subjected to SDS-PAGE and western blotting.

**Table 1:** Composition of samples for phosphatase treatment.

Condition	Cell lysates	NEB PMP buffer*	MnCl_2_*	λ PP	EDTA*	ddH_2_O	Total
**untreated**	34 μL	1×	1 mM	-	-	Up to 50 μL	50 μL
**λ PP**	34 μL	1×	1 mM	400 U	-	Up to 50 μL	50 μL
**λ PP + Na**_**3**_ **VO**_**4**_	34 μL	1×	1 mM	400 U	50 mM	-	50 μL

* Final concentration.

### Growth assay

Cells were plated at a density of 2×10^3^ cells per well in 96-well plates in D-MEM/Ham’s F-12 medium in triplicates on day 0 and counted every 24 hours over a 3-day period. Three wells at each time point were counted.

### Immunostaining

For immunostaining, cells were passaged onto 12 mm or 24 mm collagen-coated cover glasses (IWAKI), or 22 mm collagen-coated cover glasses (Corning, New York, USA). Cells were fixed with ice-cold methanol (FUJIFILM Wako) at –20°C or 2% (w/v) PFA (paraformaldehyde; Thermo Fisher) at room temperature. The fixed cells were permeabilized with 1× PEM containing 0.25% (v/v) Triton X-100 for 10 min and incubated in the blocking buffer (3% BSA in 1× PEM) for 60 min. Samples were then incubated with primary antibodies for 1 hour at room temperature or overnight at 4°C. After washing, the samples were incubated with the secondary antibodies for 1 hour at room temperature. DAPI (4’,6-diamidino-2-phenylindole; DOJINDO, Kumamoto, Japan) was added to visualize DNA when necessary. For immunostaining of cells expressing EGFP (enhanced green fluorescent protein)-tagged CAMSAP2 or CAMSAP3, plasmids were transfected 1 day before fixation, followed by similar steps as described above.

**Fig 1:**
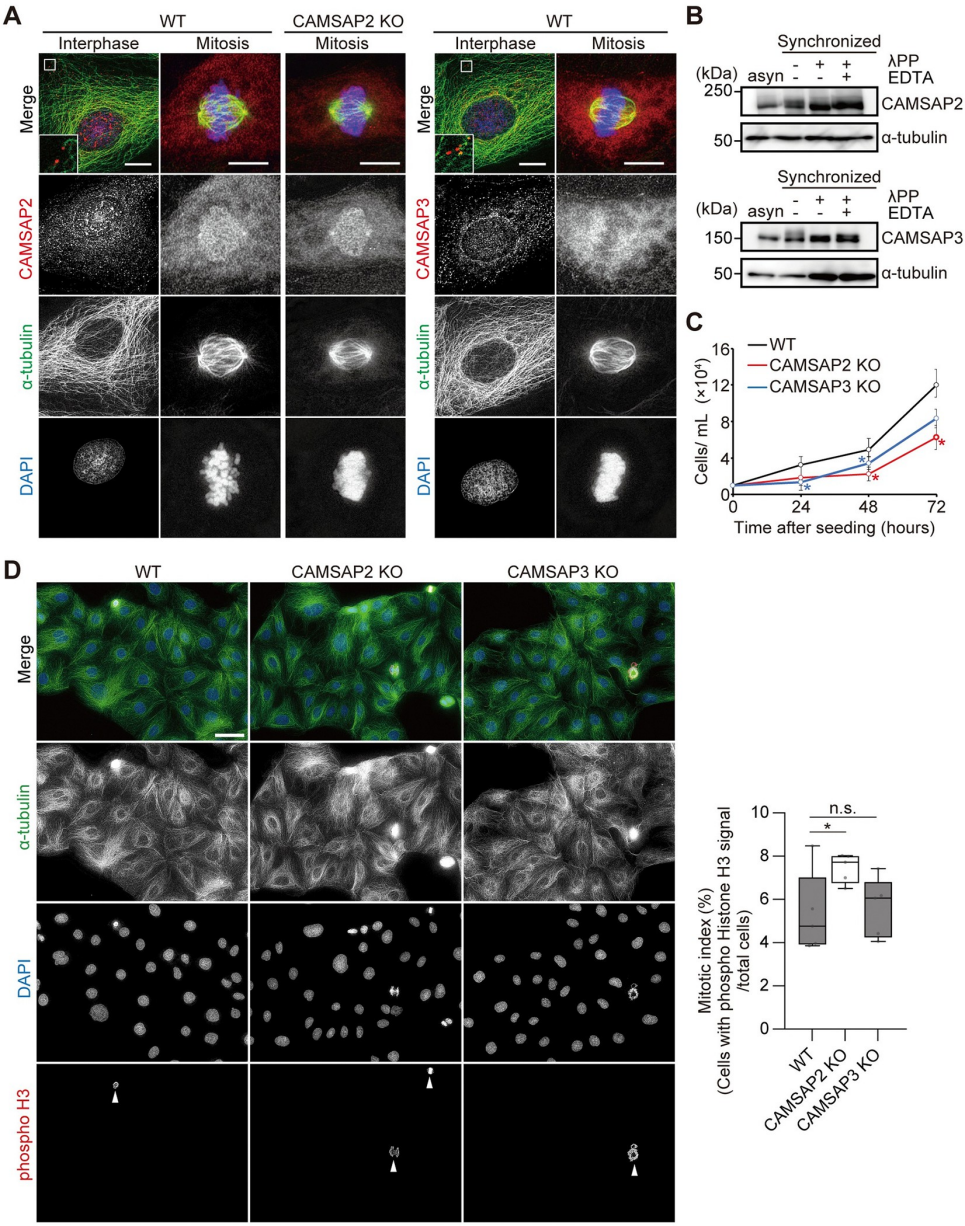
Mitotic delay in CAMSAP2 knockout Caco-2 cells. (A) Endogenous localization of CAMSAP2 and CAMSAP3 in Caco-2 cells. Cells were fixed with methanol and stained for CAMSAP2 or CAMSAP3 (red), α-tubulin (green), and DAPI (blue). Boxed regions have been enlarged, brightness-adjusted, and shown in insets to visualize CAMSAP2 and CAMSAP3 punctae at the end of microtubules. Scale bars; 10 μm. (B) Western blots showing mitotic phosphorylation of CAMSAP2 and CAMSAP3. Caco-2 WT cells were synchronized with STLC. Cell homogenates were treated with or without protein phosphatase (λPP) and with or without its inhibitor EDTA. Asynchronized cell lysates were loaded in the far left. Blots with α-tubulin are shown as loading controls. MW, kDa. (C) Growth curves for wild type (WT), CAMSAP2 knockout (KO), and CAMSAP3 KO cells. Cell concentrations at indicated hours after cell seeding were counted. Mean ± SD of three biological replicates is shown at each timepoint. *P < 0.05, Student’s t-test. Three independent experiments were performed, and the representative is shown. (D) Mitotic index of WT, CAMSAP2 KO, and CAMSAP3 KO cells. Cells were fixed with 2% PFA (paraformaldehyde) and stained for α-tubulin (green), phospho histone H3 (red), and DAPI (blue). Arrowheads indicate phospho histone H3 positive chromosomes. Percentages of cells with phospho histone H3 signals in the population were calculated as mitotic index. n = 431–638 cells per experiment, five independent experiments. Boxplots indicate 25th percentile, median, and 75th percentile values. *P < 0.05, n.s.: P > 0.05, Student’s t-test. Scale bar; 20 μm.

The following primary antibodies were used: rabbit anti-CAMSAP3 [20], a gift from Masatoshi Takeichi (RIKEN, Kobe, Japan), rabbit polyclonal anti-CAMSAP2 (Novus biologicals, Colorado, USA), mouse monoclonal anti-GFP (Roche, Basel, Switzerland), mouse monoclonal anti-γ-tubulin (GTU-88; Sigma-Aldrich), anti-human centromere protein (CREST patient serum; Antibodies Inc, California, USA), anti-KIF2A rabbit polyclonal (Novus biologicals), anti-Mad2 rabbit polyclonal (Bethyl Laboratories, Texas, USA), rabbit monoclonal (for Fig 1D) and mouse monoclonal (Fig 3A) anti-phospho histone H3 (abcam, Cambridge, UK), rabbit monoclonal anti-HAUS6 (abcam), mouse anti-Human BUBR1 (BD Transduction Laboratories™, California, USA), and rabbit polyclonal anti-CAMSAP1 (Novus biologicals).

Microtubules were stained with FITC-conjugated mouse monoclonal anti-α-tubulin (DM1A; Sigma-Aldrich) or Cy3-conjugated mouse monoclonal anti-β-tubulin antibody (clone TUB 2.1; Sigma-Aldrich).

### Microscopy

Images were acquired using a BZ-X710 Fluorescence Microscope (KEYENCE, Osaka, Japan), LSM980 laser scanning confocal microscope (Zeiss, Oberkochen, Germany), or DeltaVision-SoftWoRx system (Applied Precision, Issaquah, WA, USA). For BZ-X710, the objective lenses used were Nikon CFI Plan Apo λ 2 ×, 10 ×, 40 ×, 100 × and Nikon CFI Plan Fluor 4 ×. The acquired images were processed using BZ-X Analyzer software. For super-resolution imaging, stacks of images were captured along the z-axis at optimal intervals using Airyscan with 63x/1.4 Oil objective lenses. The acquired images were projected onto a single image by using the maximum intensity algorithm in ImageJ/Fiji. For the DeltaVision-SoftWoRx system, the acquired images were deconvoluted and projected onto a single image with a maximum intensity algorithm using SoftWoRx software (v3.7.0 and v.6.5.1) unless otherwise noted.

### Image analyses

To measure the spindle length, spindles with Mad2- or BUBR1-negative kinetochores were exclusively chosen as metaphase cells to avoid the possibility that the individual difference in the spindle length was due to the difference of mitotic stages. The spindle length defined by α- or β-tubulin signals was measured from one to the other distal edges of the spindle.

To measure the areas of the chromosomal masses, the outlines of the chromosomal masses were traced using the freehand tool in ImageJ/Fiji.

To measure the extent of displaced centrosomes, the distance between two centrosomes judged from position of a γ-tubulin dot signals was measured (γ-tubulin distance). The spindle length was measured from one to the other edges of the spindle defined by α-tubulin signals (α-tubulin distance). The degree of displaced centrosome displacement into the spindle was calculated as the ratio of the γ-tubulin distance to the α-tubulin distance.

To measure the length of the astral microtubules, images taken with LSM980 Airyscan were used. Detectable astral microtubules were traced with segmented lines and their length was measured using ImageJ/Fiji. The number of astral microtubules was counted manually.

To analyze the orientation of the spindle microtubules, images taken with LSM980 Airyscan were used. Detectable microtubules emanated from the spindle pole toward the chromosomal region were traced, in which astral microtubules were not included. The angles of the traced microtubules at the origin (spindle poles) were measured using ImageJ/Fiji software. The values were analyzed using the polar histogram algorithm in MATLAB to draw radar charts. Similar analyses were performed exclusively with astral microtubules to determine their orientation of astral microtubules.

To measure fluorescence intensity in images taken with DeltaVision-SoftWoRx system (except for bridging fibers and kinetochore fibers), images of 18 sections (in general) or 16 sections (for CAMSAP1 intensity) along the z-axis were acquired at 0.2 μm interval and projected into a single plane with sum slice algorithm without deconvolution. The background intensity was subtracted from all intensity measurements.

For α-tubulin intensity of the spindle, intensities along the spindle axis were measured using ImageJ/Fiji. Mad2-negative spindles were exclusively chosen for analysis to collect metaphase spindles. For α-tubulin intensities of the spindle in cold stable assays, the whole intensity excluding two circles of a 8-pixel diameter around spindle poles was measured using ImageJ/Fiji. For γ-tubulin intensity in the spindle, the whole intensity excluding two circles of a 8-pixel diameter around spindle poles was measured using ImageJ/Fiji. For CAMSAP1 intensity along the spindle, signals in the region of interest (ROI) surrounding the spindle were measured using ImageJ/Fiji.

To count the number of bridging fibers, images were acquired along the z-axis at optimal intervals using an LSM980 Airyscan. Each plane without z-projection was used to manually count the microtubules underlying the sister kinetochore fibers.

To measure the α-tubulin intensity of bridging and kinetochore fibers, i images were acquired along the z-axis at optimal intervals using LSM980 Airyscan. Each plane without z-projection was used to manually count the microtubules. The intensity was measured as previously reported, [14] with some modifications. For bridging fibers, a 5×5-pixel square region was selected on the center of a bridging fiber between two sister kinetochores, and its intensity was measured. For kinetochore fibers, a 5×5-pixel square region was selected next to the punctate CREST signal corresponding to the kinetochores and measured for intensity. For both fibers, the background intensity outside the spindle region was similarly measured, and its average was subtracted from the original values of the bridging or kinetochore fibers.

### Live-cell imaging

For live-cell imaging, cells were passaged in 27 mm collagen-coated glass-bottom dishes (IWAKI, Shizuoka, Japan) for 1–2 days before observation. DNA was stained with 250 ng/mL Hoechst 33342 (Invitrogen) and microtubules were stained with 4 nM SiR-tubulin (Spirochrome, Stein am Rhein, Germany) supplemented with 10 μM Verapamil depending on experimental conditions. Before observation, cells were incubated in D-MEM/Ham’s F-12 medium containing Hoechst and/or SiR-tubulin with Verapamil for 30 min at 37°C in 5% CO_2_. The medium was replaced with Leibovitz’s L-15 Medium without Hoechst, SiR-tubulin, and Verapamil right before observation. Live-cell imaging was performed with the DeltaVision-SoftWoRx system.

For CAMSAP1 localization, WT and CAMSAP2 KO cells were transfected with plasmids (see above) 1 day before observation. DNA and microtubules were stained as described previously. To analyze chromosome segregation, WT and CAMSAP2 KO cells were prepared for live-cell imaging with 250 ng/mL Hoechst 33342. Images of eight sections along the z-axis were acquired at 0.8-μm intervals every 30 seconds. The timing of “first alignment,” “anaphase onset,” and “completion of chromosome segregation” was defined as follows: first alignment was defined as the time point when chromosomes first aligned in the equatorial plane. Anaphase onset was defined as the time point at which the chromosomes began to distribute toward the poles. The completion of chromosome separation was defined as the time point at which chromosome clusters moving to the poles were completely separated from each other.

To analyze spindle elongation, WT and CAMSAP2 KO cells expressing EB1-mCherry2 at the endogenous levels were prepared for time-lapse imaging using Hoechst. Images of eight sections along the z-axis were acquired at 1.2-μm intervals every 20 seconds. Spindle length was measured as the distance between the two spindle poles every 20 seconds. The spindle length at each time point was normalized to the spindle length at anaphase onset (defined as t = 0). The average speed of spindle elongation was calculated based on the change in spindle length during the observation period (300 seconds after the onset of anaphase). The spindle elongation speed during observation was calculated every 20 seconds: v = Δ(spindle length) / Δt (20 sec) and shown in Fig 5H (bottom) and S2 Table.

### Western blotting

Cell lysates were collected with a 1× sample buffer. Proteins were separated in 5–20% (w/v) gradient SDS-PAGE precast gels (FUJIFILM Wako) or in-house 10 or 6% SDS-PAGE gels. Proteins run in gels were transferred to PVDF membranes (Millipore, Billerica, Massachusetts, USA) using a wet-tank system. Membranes were blocked with 3% (w/v) skim milk in TBS with 0.1% Tween 20 (TBS-T) for 60 min and incubated with primary antibodies for 1 hour at room temperature or overnight at 4°C. The membranes were washed and incubated with secondary antibodies for 1 hour at room temperature. Proteins were detected using Luminata Forte Western HRP substrate (Millipore). For multiple detections with different antibodies on the same membranes, blotted membranes were soaked in Stripping Solution (FUJIFILM Wako) for 10 min at room temperature or solution of 1× TBS-T including 2% SDS and 0.05% β-mercaptoethanol (nacalai tesque, Kyoto, Japan) for 50 min at 30°C and washed four times with TBS-T for 5 min each time. Primary antibodies used for western blotting were as follows: Rabbit anti-CAMSAP3 (a gift from Masatoshi Takeichi [20]; SIGMA ALDRICH), rabbit polyclonal anti-CAMSAP2 (Novus biologicals, Colorado, USA), anti-KIF2A rabbit polyclonal (Novus biologicals), mouse anti-GAPDH antibody (MBL, Tokyo, Japan), rabbit monoclonal anti-HAUS6 (abcam), rabbit polyclonal anti-CAMSAP1 (Novus biologicals), mouse monoclonal anti-γ-tubulin (GTU-88; Sigma-Aldrich), and mouse monoclonal anti-α-tubulin (DM1A; Sigma-Aldrich). Secondary antibodies used were as follows: anti-rabbit IgG HRP-linked whole (Cytiva, Tokyo, Japan) and Peroxidase AffiniPure Sheep Anti-Mouse IgG (H+L) (Jackson ImmunoResearch, Pennsylvania, USA). For chemiluminescence imaging, the CCD imager LAS 500 (GE Healthcare) was used for all the blots except for the blot for CAMSAP3 KO (S5B Fig) in which LAS 3000 was used. Band intensities were measured using ImageJ/Fiji.

### Cold stable assay

A metal plate, medium, and fixation solution were cooled on ice prior to use. Ice-cold medium was added to the cells, which were then placed on an ice-cold metal plate. After 7 min, cells were fixed with 2% PFA on an ice-cold metal plate. Subsequent steps were similar to those used for standard immunostaining at room temperature. Control samples were prepared by performing all the steps at room temperature.

### FACS analysis

Cultured Caco-2 cells were stripped from the dishes and collected using 0.05% trypsinization. The cells were then centrifuged at 300 ×g for 5 min and washed twice with PBS. Cell pellets were suspended in 70% ethanol at –20°C and fixed overnight at 4°C. After fixation, cells were centrifuged at 300 ×g for 5 min and washed twice with PBS. Subsequently, 0.25 mg/ml RNase A solution (QIAGEN, Holland) was added to approximately 1 × 10^6^ cells and the samples were allowed to stand at 37°C for 20 min. Propidium iodide solution (Wako) was added at a final concentration of 50 μg/mL and the samples were allowed to stand in the dark place at 4°C for 30 min. Cells were filtered through a 40-μm cell strainer and analyzed using a cell analyzer Cytomics FC500MPL. Notably, 10000 cells per sample were used for the analyses.

### Statistics

Radar charts were created using a polar histogram algorithm in MATLAB (MathWorks, Massachusetts, USA). All boxplots shown in the figures were prepared using GraphPad Prism 9 (version 9.4.1 (458); GraphPad Software, Boston, MA) and created according to the following definition. The centerline indicates the median value. The top edge of the box indicates the 25th percentile and the bottom edge of the box indicates the 75th percentile, which were determined using R software. Whiskers indicate the range between the maximum and minimum values. The statistical tests used in the experiments are presented in the figure legends.

## Results

### Mitotic delay in CAMSAP2 KO Caco-2 cells

In Caco-2 cells, a human epithelial cell line, microtubules are mainly non-centrosomal in interphase, and CAMSAP2 and CAMSAP3 are known to be associated with the minus ends of microtubules to stabilize them [19,20], as their microtubule-end localization was observed in Caco-2 cells immunostained with anti-CAMSAP2 and anti-CAMSAP3 antibodies (Fig 1A; S1 and S2 Figs). When cells entered mitosis, as previously reported in HeLa cells [21], CAMSAP2 and CAMSAP3 localization became undetectable (Fig 1A; S1 and S2 Figs). Although anti-CAMSAP2 staining of Caco-2 WT cells in prometa–metaphase showed non-specific fluorescence signals at the spindle poles, centrosomal signals were also observed in CAMSAP2 KO Caco-2 cells stained with the anti-CAMSAP2 antibody (S1B Fig). Dispersal of mitotic CAMSAP2 was similarly observed in cells overexpressing N-terminally EGFP-tagged CAMSAP2, as visualized using anti-GFP antibodies (S3A Fig). Lack of CAMSAP3 signals at the spindle poles in immunostaining (S2A Fig) was also confirmed by EGFP-CAMSAP3 episomal expression followed by immunostaining. But when EGFP-CAMSAP3 was episomally expressed, immunostaining with an anti-GFP antibody occasionally indicated signals at the spindle poles (S3B Fig). This was mostly owing to overexpression, as cells with low EGFP-CAMSAP3 expression did not show signals at the poles. Therefore, we confirmed that both CAMSAP proteins were diffusely expressed in metaphase cells. Similar to that observed in HeLa cells [21], western blots of lysates prepared from Caco-2 cells asynchronized or synchronized in mitosis (S4 Fig) showed that the CAMSAP2 and CAMSAP3 bands of mitotic cells were shifted up compared to those of asynchronous cells (Fig 1B). The band shift was regressed when λ-phosphatase was added; however, the same was not observed when λ-phosphatase was added together with its inhibitor EDTA (Fig 1B), suggesting that the phosphorylation state of CAMSAP proteins may reflect their diffusive localization during mitosis. Therefore, CAMSAP2 and CAMSAP3 in both HeLa [21,41] and Caco-2 cells are considered to function in interphase through their localization to the minus ends of microtubules. However, the same is not observed in mitosis, as these proteins may be regulated in mitosis to lose their localization through phosphorylation. Therefore, little attention has been paid to their functions in mitosis.

In contrast, Patronin, a *Drosophila* ortholog of the CAMSAP/Patronin family, localizes to spindle poles during mitosis to maintain spindle length and proper chromosome segregation [37–39]. In the absence of Patronin, the minus ends of microtubules in both the mitotic spindle and interphase cytoplasmic microtubules are destabilized by kinesin-13, which depolymerizes microtubules, leading to shortening of the mitotic spindle and a sparse array of interphase microtubules [38]. In our study using Caco-2 cells for intestinal functions of the CAMSAP3 protein [26], we realized that both CAMSAP3 KO and CAMSAP2 KO cells created in this study (S5A and S5B Fig) showed slow growth in culture. Growth curve assays suggested that both CAMSAP2 KO and CAMSAP3 KO cells were slow in proliferation (Fig 1C). Although FACS analyses detected no obvious delays in the G1–S–G2 phases of the cell cycle (S5C Fig), the mitotic index calculated by staining of phosphorylated histone H3, indicative of condensed mitotic chromatin [46], revealed that CAMSAP2 KO cells showed higher percentages of mitotic cells than WT cells (Fig 1D). In contrast, CAMSAP3 KO cells exhibited an almost normal mitotic index that was similar to WT cells (Fig 1D), suggesting that the growth defects of CAMSAP3 KO cells are derived from undetermined abnormalities, except for cell cycle-dependent ones. Therefore, CAMSAP2, but not CAMSAP3, may have an unexplored function during mitosis, which led us to further examine the mitotic phenotype of CAMSAP2 KO cells.

### CAMSAP2 KO results in short spindle with displaced spindle poles and sparse midzone microtubules

**Fig 2:**
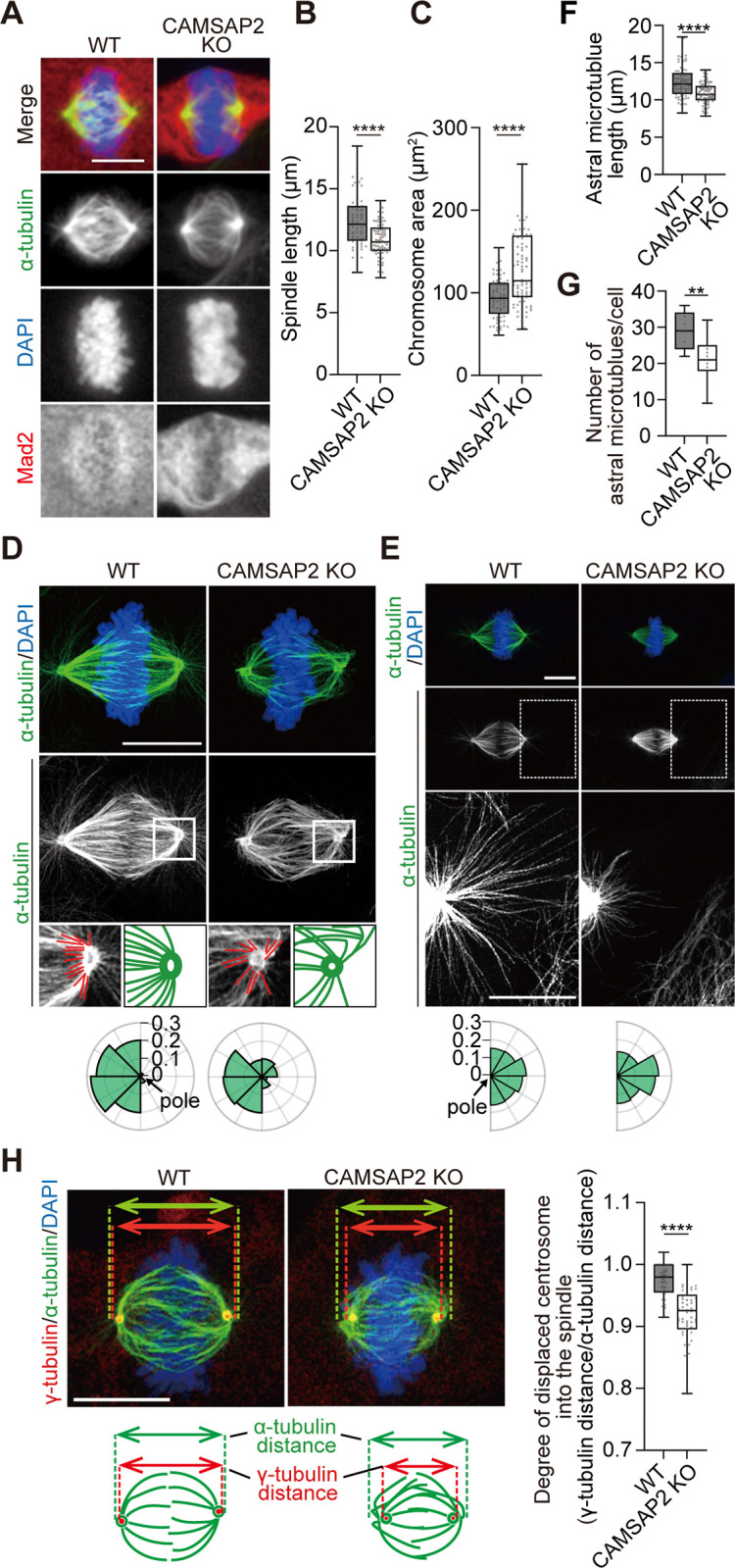
Defective spindle morphology in CAMSAP2 KO cells. (A) Representative images of WT and CAMSAP2 KO spindles. Notably, 2% PFA-fixed cells were stained for α-tubulin (green), Mad2 (red), and DAPI (blue). Scale bar; 10 μm. (B) The spindle length of cells prepared in (A) measured based on α-tubulin signals. Mad2-negative spindles were exclusively chosen for quantification as of metaphase. n = 79 (WT) and 86 (CAMSAP2 KO) cells from four independent experiments were analyzed. Boxplots indicate 25th percentile, median, and 75th percentile values. ****P < 0.0001, Welch’s t-test. (C) Areas of chromosomes in metaphase cells prepared in (A), measured from DAPI signals. Mad2-negative spindles were exclusively chosen for quantification. n = 79 (WT) and 86 (CAMSAP2 KO) cells from four independent experiments. Boxplots indicate 25th percentile, median, and 75th percentile values. ****P < 0.0001, Welch’s t-test. (D) Orientation of polar microtubules extending inward from the spindle. Representative images acquired with the super-resolution confocal microscope LSM980 Airyscan are shown. Cells were fixed with 2% PFA and stained for α-tubulin (green) and DAPI (blue). The centrosome regions are boxed and enlarged below. Radar charts show distribution of angles of polar microtubules emanating towards chromosomes. Astral microtubules are not included. The origin of microtubules (the spindle pole) was centered in the chart and labels on the radar axis indicate frequencies. The sum of all the frequencies equals 1.0. Each microtubule emanating from the pole (traced in red) is taken to measure its orientation. n = 158 microtubules from 9 cells (WT) and 139 microtubules from 9 cells (CAMSAP2 KO) from four independent experiments. Scale bar; 10 μm. (E) Orientation of astral microtubules as in (D). Boxed regions are enlarged and brightness is adjusted to visualize astral microtubules. Radar charts show distribution of angles of astral microtubules. n = 255 microtubules from 11 cells (WT) and 336 from 12 cells (CAMSAP2 KO) from four independent experiments. Scale bars; 10 μm. (F, G) The length (F) and number (G) of astral microtubules captured in (E) are quantified. For (F), n = 263 microtubules from 9 cells (WT) and 324 from 15 cells (CAMSAP2 KO) from three independent experiments. Boxplots indicate 25th percentile, median, and 75th percentile values. ****P < 0.0001, Welch’s t-test. For (G), n = 9 cells (WT) and 15 cells (CAMSAP2 KO) from three independent experiments. **P < 0.01, Student’s t-test. (H) Definition of the degree of displaced centrosomes. Cells were fixed with 2% PFA and stained for α-tubulin (green), γ-tubulin (red), and DAPI (blue). The γ-tubulin punctate signals were defined as centrosomes, and the distance between two γ-tubulin dots was defined as the γ-tubulin distance (red bidirectional arrows). The distance between two α-tubulin signals at the tips of the spindle was defined as the α-tubulin distance (green bidirectional arrows). Degree of displaced centrosome was calculated as the ratio of γ-tubulin distance to α-tubulin distance. n = 57 (WT) and 59 (CAMSAP2 KO) cells from three independent experiments. Boxplots indicate 25th, median, and 75th percentile values. ****P < 0.0001, Welch’s t-test. Scale bar; 10 μm.

We examined the spindle morphology of CAMSAP2 KO cells by immunostaining microtubules. The length of metaphase spindles was significantly shorter in CAMSAP2 KO cells (10.88 ± 0.15 μm) than that in WT cells (12.44 ± 0.25 μm) (Fig 2A and 2B). To precisely pinpoint metaphase spindles for quantification of length, we co-stained Mad2 (or BubR1 depending upon the case), the component(s) of the spindle assembly checkpoint, and chose the spindle in which Mad2 (or BubR1) did not localize to kinetochores. Spindle length was not necessarily proportional to cell size, as no correlation was found between spindle length and cell diameter in either WT or CAMSAP2 KO cells (S6 Fig).

The alignment of kinetochores at the spindle equator, judged from the area of the chromosome mass, was less organized in CAMSAP2 KO than in WT cells (Fig 2A and 2C). In addition, microtubules within the spindle midzone were sparse in CAMSAP2 KO metaphase cells (Fig 2A and 2D; see below). Super-resolution imaging revealed that microtubules in the vicinity of the spindle poles (excluding astral microtubules) were disrupted in CAMSAP2 KO cells (Fig 2D). In WT cells, spindle microtubules around the spindle poles were oriented towards the equatorial plane, whereas in CAMSAP2 KO cells, some microtubules did not orient towards the equatorial plane and were oriented away from the spindle pole and then toward the center of the spindle (Fig 2D). In contrast, the orientation of the astral microtubules was similar in the WT and CAMSAP2 KO cells (Fig 2E), although the length and number of astral microtubules were reduced in the CAMSAP2 KO cells (Fig 2E–2G). Therefore, it is possible that CAMSAP2 is essential for the stabilization of astral microtubules; however, not for their orientation, although CAMSAP2 is required for the correct orientation of interpolar microtubules.

Co-staining microtubules with γ-tubulin, a marker for centrosome, showed that centrosomes were observed displaced inside the mitotic spindle in CAMSAP2 KO cells (Fig 2H). Therefore, CAMSAP2 KO cells showed defects in spindle morphology, that is, short spindles, as well as a lack of integrity in microtubule organization at the spindle poles and midzone. The phenotypes of CAMSAP2 KO, such as short spindles and displaced centrosomes, were rescued by the episomal expression of GFP-CAMSAP2 (S7A Fig).

Next, we examined whether the spindle phenotype of CAMSAP2 KO cells was due to alterations in other CAMSAP proteins. As CAMSAP1 is known to localize to spindle microtubules [40], we examined whether the phenotype of CAMSAP2 KO be attributed to a possible loss of CAMSAP1 from the spindle. The protein level of CAMSAP1 in CAMSAP2 KO was slightly decreased, albeit of no statistical significance (S7B Fig). As CAMSAP1 localized normally to the spindle in CAMSAP2 KO cells (S7C and S7D Fig), the spindle phenotype of CAMSAP2 is not mainly due to a lack of CAMSAP1, although we cannot exclude the possibility of partial contribution of CAMSAP1 reduction to the phenotype of CAMSAP2 KO cells. Regarding CAMSAP3, neither CAMSAP2 KD [20] nor KO (S7E Fig) significantly affected the protein levels, denying the possibility that the mitotic phenotype of CAMSAP2 KO was due to CAMSAP3 upregulation. CAMSAP2 localization was not altered in CAMSAP3 KO cells (S8 Fig).

### Depletion of KIF2A suppresses CAMSAP2 phenotype of mitotic delay and the short spindle, but not displaced spindle poles

**Fig 3:**
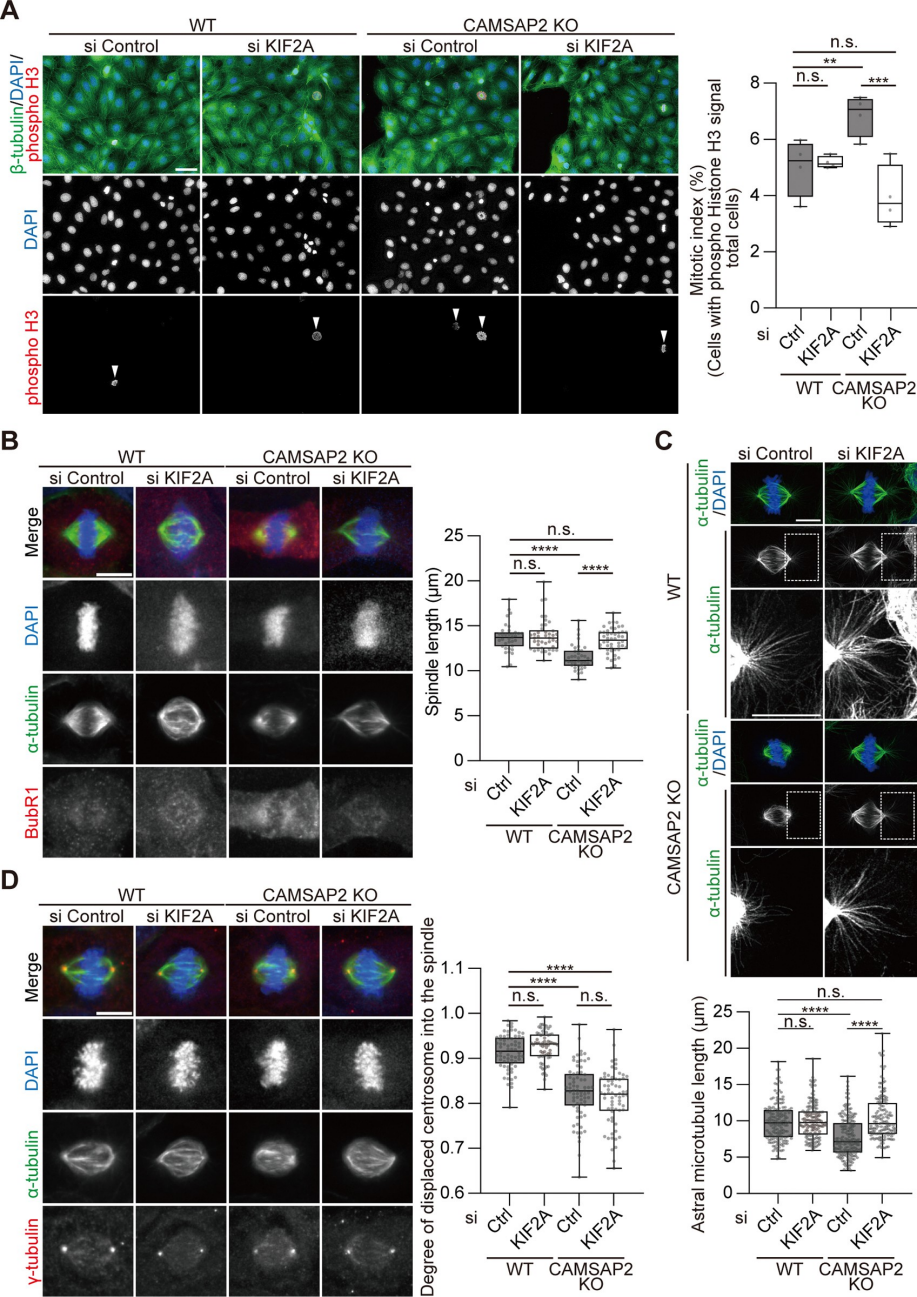
KIF2A depletion partially suppresses mitotic defects of CAMSAP2 KO cells. (A) Mitotic index of WT and CAMSAP2 KO cells with (si KIF2A) or without (si Control) KIF2A knockdown. Cells were fixed with 2% PFA and stained for β-tubulin (green), phospho histone H3 (red), and DAPI (blue). Arrowheads, chromosomes with phospho histone H3 signals. Mitotic index was measured as in Fig 1D. n = 490–786 cells per experiment, three independent experiments. Boxplots indicate 25th percentile, median, and 75th percentile values. ***P < 0.001, **P < 0.01, n.s. > 0.05, Two-way ANOVA followed by Tukey’s multiple comparison tests. Scale bar; 20 μm. (B) Spindle length in WT and CAMSAP2 KO cells with and without KIF2A knockdown. Methanol-fixed cells were stained for α-tubulin (green), BubR1 (red), and DAPI (blue). BubR1-negative spindles were exclusively chosen as metaphase cells for size measurement. n = 35 (si-control WT), 42 (si KIF2A WT), 39 (si-control CAMSAP2 KO), and 48 (si KIF2A CAMSAP2 KO) cells from three independent experiments. Boxplots indicate 25th, median, and 75th percentile values. ****P < 0.0001, n.s.: P > 0.05, Two-way ANOVA followed by Tukey’s multiple comparison tests. (C) Astral microtubules in WT and CAMSAP2 KO cells with or without KIF2A knockdown. Cells were fixed with 2% PFA and stained for α-tubulin (green) and DAPI (blue). Representative images acquired using an LSM980 Airyscan are shown. Boxed regions are enlarged below, and brightness is adjusted for visualization of astral microtubules. Scale bars; 10 μm. To quantify the length, n = 160 microtubules from 9 cells (si-control WT), 153 from 9 cells (si KIF2A WT), 181 from 8 cells (si-control CAMSAP2 KO), and 144 from 7 cells (si KIF2A CAMSAP2 KO) from three independent experiments. Boxplots indicate 25th, median, and 75th percentile values. **** P < 0.0001; n.s.: P > 0.05, Two-way ANOVA followed by Tukey’s multiple comparison tests. (D) The position of centrosomes (γ-tubulin signals) in CAMSAP2 KIF2A co-depleted cells. The degree of displaced centrosomes was calculated similarly, as shown in Fig 2H. n = 68 (si-control WT), 65 (si KIF2A WT), 72 (si-control CAMSAP2 KO), and 65 (si KIF2A CAMSAP2 KO) cells from three independent experiments. Boxplots indicate 25th, median, and 75th percentile values. ****P < 0.0001, n.s.: P > 0.05, Two-way ANOVA followed by Tukey’s multiple comparison tests.

Patronin, the CAMSAP homolog in *Drosophila*, antagonizes the kinesin-13 microtubule depolymerase Klp10A at spindle poles, thereby regulating spindle length [38,39]. The short spindle phenotype of Patronin KD was restored by simultaneous depletion of Klp10A [38]. Unlike Patronin, CAMSAP2 did not localize to spindle poles and was diffusely observed during mitosis (Figs 1A and S1). However, the short-spindle phenotype of CAMSAP2 KO cells was similar to that of Patronin-deficient cells. Therefore, we examined whether depletion of KIF2A (S9 Fig), the mammalian kinesin-13, restored the mitotic phenotype caused by CAMSAP2 KO. The increase of mitotic index in CAMSAP2 KO (si-Ctrl CAMSAP2 KO, 7.45 ± 0.30%; Fig 3A) was repressed to WT levels by simultaneous depletion of KIF2A (si-KIF2A CAMSAP2 KO, 3.90 ± 0.78%). The cancellation of the mitotic index increase could be interpreted as because of possible synthetic cell death by co-depletion. To exclude that possibility, we examined the mechanism by which KIF2A depletion suppresses the mitotic phenotype of CAMSAP2 KO. The spindle length of CAMSAP2 KO cells (11.49 ± 0.22 μm) was shorter than WT (13.72 ± 0.26 μm); however, it was restored to 13.35 ± 0.20 μm which is comparable to WT (Fig 3B). Astral microtubules were shortened in CAMSAP2 KO cells (Figs 2E and 3C); however, their length was restored by simultaneous KIF2A depletion (si-KIF2A CAMSAP2 KO, Fig 3C). The increase in astral microtubule length was not statistically significant in a single KD of KIF2A (si-KIF2A, Fig 3C). This was partly because of the underestimation of the length, as astral microtubules often reached the cell cortex, which inhibited further extension of the microtubules (Fig 3C). Therefore, the length of the astral microtubules is determined by the antagonistic roles of CAMSAP2 (stabilization) and KIF2A (depolymerization).

Notably, the CAMSAP2 KO-induced phenotype of displaced centrosomes was not suppressed by the simultaneous depletion of KIF2A (Fig 3D). Therefore, CAMSAP2 cooperates in part with KIF2A to assemble mitotic spindles to a proper size while maintaining the integrity of spindle poles separately from KIF2A.

### Non-kinetochore microtubules and bridging fibers are reduced in the CAMSAP2 KO spindle

**Fig 4:**
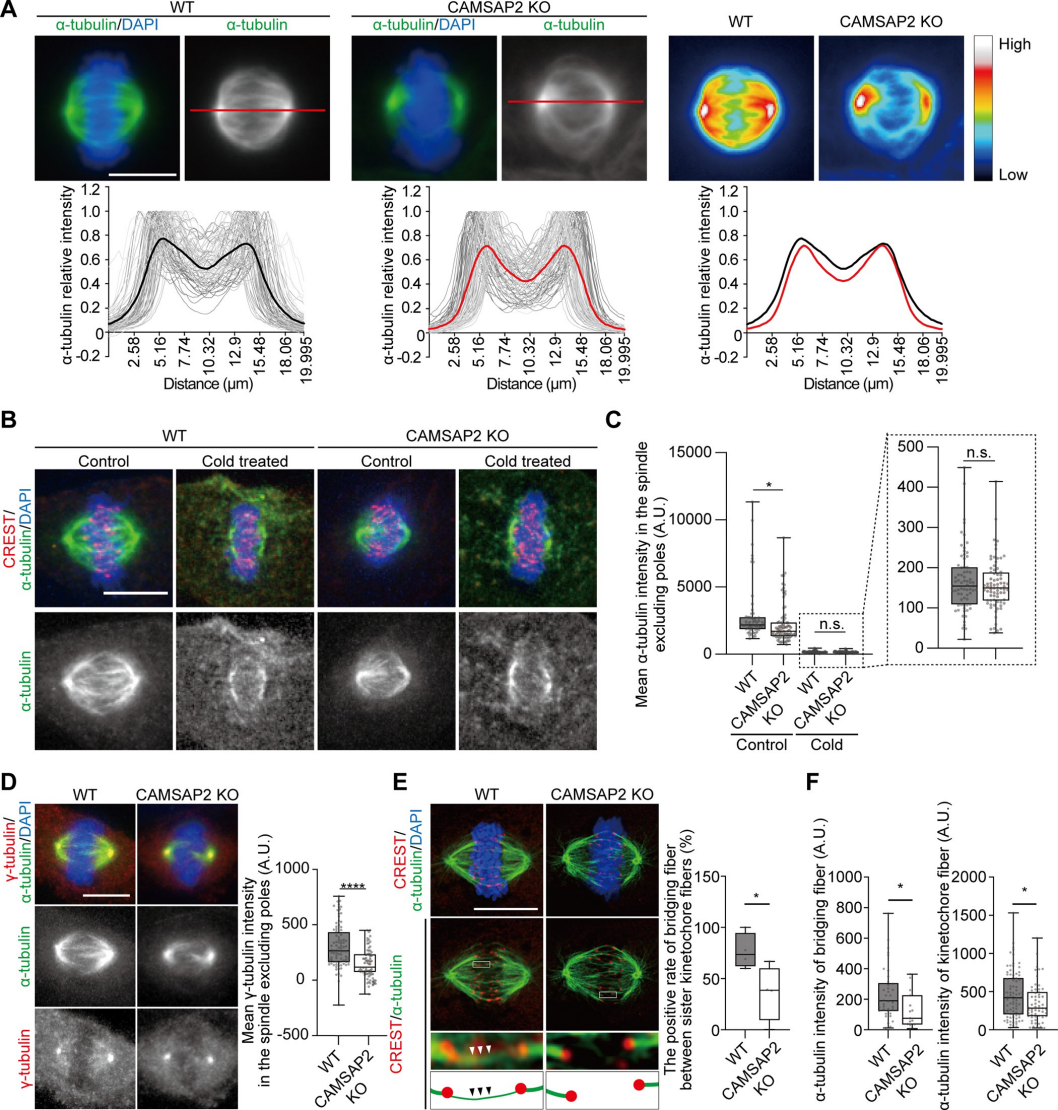
Decreased spindle microtubules in CAMSAP2 KO cells. (A) Signal intensity of α-tubulin immunostained in WT and CAMSAP2 KO cells quantified along the spindle axis (red lines). Notably, 2% PFA-fixed cells stained for α-tubulin (green) and DAPI (blue). Images analyzed in Fig 2A were used for quantification. Heatmaps indicate gradient fluorescence intensities of α-tubulin signals. Lower graphs show in gray, each spindle; black (WT) and red (CAMSAP2 KO), average of all spindles. Background intensities were subtracted, which were then normalized by the maximum values of the spindles. Mad2-negative spindles were chosen as metaphase ones for quantification. n = 79 (WT) and 86 (CAMSAP2 KO) cells from four independent experiments. Scale bar; 10 μm. (B) Representative images of spindle untreated (control) and cold treated WT and CAMSAP2 KO cells. Notably, 2% PFA-fixed cells were stained for α-tubulin (green), CREST (red), and DAPI (blue). Scale bar; 10 μm. (C) Signal intensities of α-tubulin in spindles of (B). The mean intensity in the spindle excluding poles were measured. The boxed region is shown magnified to the right. The background intensity off the spindle was subtracted. n = 57 (control WT), 71 (cold treated WT), 83 (control CAMSAP2 KO), and 76 (cold treated CAMSAP2 KO) cells from three independent experiments. Boxplots indicate 25th percentile, median, and 75th percentile values. *P < 0.05, control WT vs cold treated WT: Welch’s t-test, n.s.: P > 0.05, control CAMSAP2 KO vs cold treated CAMSAP2 KO: Student’s t-test. (D) γ-tubulin intensities on the spindle. Notably, 2% PFA-fixed cells were stained for α-tubulin (green), γ-tubulin (red), and DAPI (blue). γ-tubulin signal intensities on the spindle excluding the poles were quantified. The background intensity off the spindle was subtracted. n = 88 (WT) and 76 (CAMSAP2 KO) cells from three independent experiments. Boxplots indicate 25th percentile, median, and 75th percentile values. ****P < 0.0001, Welch’s t-test, Scale bar; 10 μm. (E) Visualization of bridging fibers in the spindle. Representative images of single z-planes acquired with LSM980 Airyscan are shown. Notably, 2% PFA-fixed cells were stained for α-tubulin (green), CREST (red), and DAPI (blue). Insets are enlarged with adjusted brightness, showing sister kinetochore fibers and bridging fibers behind the sister-kinetochore fibers (arrowheads). Frequencies of sister kinetochore pairs accompanying bridging fibers behind. n = 56 pairs of kinetochores from 18 cells (WT) and 53 from 14 cells (CAMSAP2 KO) from four independent experiments. Boxplots indicate 25th, median, and 75th percentile values. *P < 0.05, Student’s t-test. Scale bar; 10 μm. (F) α-tubulin intensity of bridging fibers (left) and kinetochore fibers (right). The intensity was measured according to a previous report with some modifications [14]. For the intensities of bridging fibers (left), 38 fibers from 17 cells (WT) and 15 from 10 cells (CAMSAP2 KO) were obtained from three independent experiments. Pairs without visible bridging fibers were excluded from quantification. For kinetochore fibers (right), 86 kinetochore fibers from 17 cells (WT) and 59 from 13 cells (CAMSAP2 KO) from three independent experiments. Boxplots indicate 25th, median, and 75th percentile values. *P < 0.05, Student’s t-test.

As mentioned above, microtubules were sparsely observed in the mid-zone of the mitotic spindle in CAMSAP2 KO cells (Figs 2 and 4A). Measurements of the intensity for α-tubulin immunofluorescence signal suggested that the number of microtubules was reduced in CAMSAP2 KO spindles, particularly in the midplane of the metaphase spindles, where the chromosomes were aligned (Fig 4A). Microtubules that constitute the spindle are classified into several types based on their roles and directionality/interactions, such as astral microtubules, kinetochore microtubules, and non-kinetochore microtubules [1–4]. Not all the microtubules are nucleated from centrosome (centrosomal microtubules). Instead, some microtubules are nucleated along the existing microtubules (microtubule-based microtubules) and are part of them [8–12]. We examined which class of microtubules was affected in mitotic spindles after CAMSAP2 KO. We first tested whether kinetochore microtubules were affected in the CAMSAP2 KO spindles. To this end, a cold stable assay was performed. Microtubules generally depolymerize at low temperatures; however, stable microtubules such as kinetochore microtubules are less likely to depolymerize at low temperatures [47]. Before the cold treatment, α-tubulin intensities of the spindle were higher in WT than in CASMSAP2 KO (Fig 4B and 4C). After the cold treatment, α-tubulin signals decreased to similar levels in WT and CAMSAP2 KO, reflecting that astral and non-kinetochore microtubules had undergone depolymerization, and that the amount of remaining ones, corresponding to kinetochore-microtubules, was comparable in WT and CAMSAP2 KO cells (Fig 4B and 4C). These results suggest that the microtubules reduced within the CAMSAP2 KO spindles are not kinetochore microtubules, but non-kinetochore microtubules around the midplane.

Non-kinetochore microtubules include both centrosomal and non-centrosomal microtubules. In interphase Caco-2 cells, centrosomal microtubules became predominant after CAMSAP2 depletion [20]. Therefore, we examined whether non-centrosomal microtubules within the spindle were affected by CAMSAP2 KO. Within the mitotic spindles, the microtubule nucleator γ-TuRC is recruited to the walls of existing microtubules by the γ-tubulin recruiting complex Augmin, which “amplifies” the microtubules within spindle, independent of the centrosome [8,10–12]. In WT spindles, γ-tubulin signal was observed throughout the spindles with a signal intensity of 307.61 ± 20.39 A.U., whereas in CAMSAP2 KO spindles the γ-tubulin signal was significantly reduced to 155.95 ± 14.16 A.U. (Fig 4D). Western blotting revealed that protein levels of neither γ-tubulin nor HAUS6 (a component of the Augmin complex) were significantly altered under CAMSAP2 KO conditions (S10A and S10B Fig). These results indicate that γ-tubulin is not down-regulated, and the reduction of γ-tubulin is mainly on non-centrosomal microtubules. This is consistent with a previous observation that centrosomal microtubules are predominant to non-centrosomal microtubules in CAMSAP2 KO cells in interphase [20]. At the minus-ends of microtubule, the binding of γ-TuRC and CAMSAP proteins is mutually exclusive [21,25]. In addition, CAMSAP2 was diffusely observed throughout the mitotic cells (Figs 1A and S1A). These observations suggest that CAMSAP2 facilitates the formation of Augmin-induced microtubules, that is, microtubules nucleate along existing spindle microtubules, even though localization of CAMSAP2 to the spindle was not observed.

A recent report showed that the Augmin complex is involved in the formation of spindle microtubule arrangements consisting of two sister kinetochore microtubules connected by a bridging fiber [14]. Depletion of a component of the Augmin complex inhibits the nucleation of bridging fibers and impairs mitotic fidelity. Our observation, using super-resolution microscope, showed that 76.75 ± 8.56% of spindles in WT cells had bridging fibers connecting the microtubules of the two sister kinetochores, whereas in CAMSAP2 KO, the number of spindles containing bridging fibers decreased to 35.91 ± 13.69% (Fig 4E). The reduction of α-tubulin intensities was seen in bridging fibers as well as in kinetochore fibers (Fig 4F). These results suggest that Augmin-mediated bridging fibers are impaired in the mitotic spindles of CAMSAP2 KO cells.

To investigate the possibility that CAMSAP2 facilitates spindle assembly through the Augmin pathway, single and double depletion of CAMSAP2 and HAUS6 was performed (S10C Fig). The spindle length of the co-depleted cells (si-HAUS6 CAMSAP2 KO, S10D Fig) was slightly shorter than that of cells with a single depletion. This reduction might indicate that CAMSAP2 plays a role in spindle sizing largely through the Augmin pathway (bridging fiber assembly), and that Augmin contributes to spindle sizing in a CAMSAP2-independent manner. Augmin assembles a wider range of spindle microtubules than CAMSAP2 does.

Subsequently, to focus on non-centrosomal microtubules, that is, bridging fibers, we first measured the amount of γ-tubulin that localizes to the spindle excluding the poles. γ-tubulin levels were reduced in HAUS6 single depletion as well as in CAMSAP2 HAUS6 double depletion to a similar degree (S10E Fig). Finally, the reduction in bridging fibers in CAMSAP2 HAUS6 double depletion was comparable to that in HAUS6 single depletion (S10F Fig). As there were no additive effects of CAMSAP2 and HAUS6 KD on the assembly of bridging fibers, we propose that CAMSAP2 is required for spindle assembly, particularly that of bridging fibers, through the Augmin pathway.

### Chromosome mis-segregation and impaired spindle elongation in CAMSAP2 KO cells

**Fig 5:**
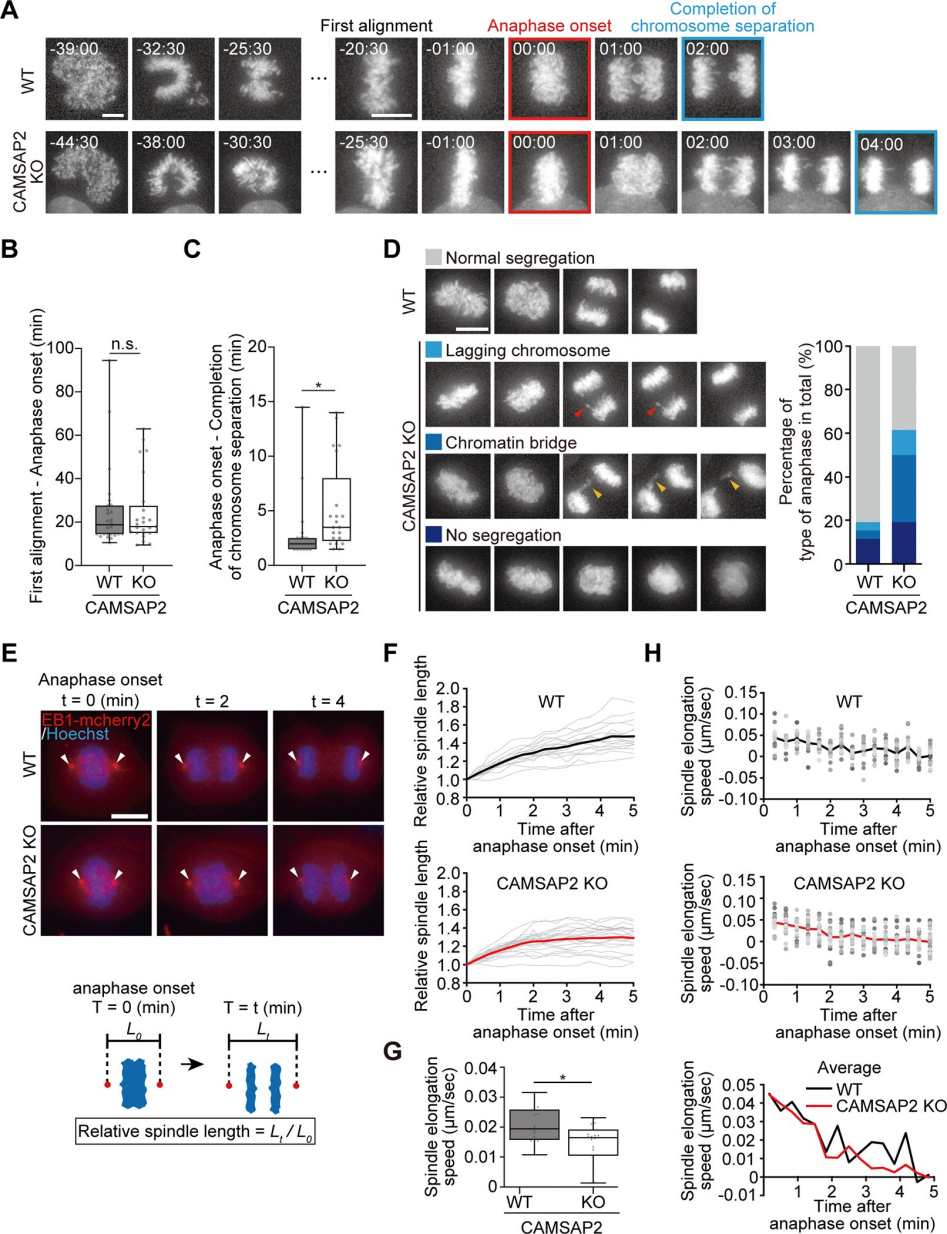
Defects in chromosome segregation with slowed anaphase spindle elongation in CAMSAP2 KO. (A) Alignment and segregation of chromosomes during mitotic progression of WT and CAMSAP2 KO cells visualized with Hoechst staining. ‘First alignment’ was defined as the timepoint when chromosomes first aligned on the equatorial plane. ‘Anaphase onset’ was defined as the timepoint when the chromosomes began to separate (t = 0). ‘Completion of chromosome separation’ was defined as the timepoint when the chromosome masses completely separated poleward. time; mm:ss, Scale bar; 10 μm. (B) Duration from first alignment to anaphase onset. n = 26 cells from eight independent experiments (WT) and 25 from nine independent experiments (CAMSAP2). (C) Duration from anaphase onset to completion of chromosome separation. Cells showing cytokinetic failures or no segregation (see D) are not included. n = 23 cells from eight independent experiments (WT) and 21 from nine independent experiments (CAMSAP2). Boxplots indicate 25th percentile, median, and 75th percentile values. *P < 0.05, Student’s t-test. (D) Patterns of chromosome segregation in WT and CAMSAP2 KO cells were classified into the indicated categories. Red arrowheads indicate lagging chromosomes and yellow arrowheads indicate a chromosomal bridge. Percentages of each category are shown in the right. n = 26 cells (WT) from eight independent experiments and 26 (CAMSAP2 KO) from nine independent experiments. Scale bar; 10 μm. (E) Time-lapse images of EB1-mCherry2 (red) expressed in WT and CAMSAP2 KO cells. DNA (blue) was stained with Hoechst. Arrowheads indicate spindle poles. Schematics designate how to calculate the relative spindle length at each timepoint. Scale bar; 10 μm. (F) Relative spindle length after anaphase onset calculated as in (E). Gray, each spindle. Black (WT) and red (CAMSAP2 KO), average of all the samples. n = 18 cells (WT) from six independent experiments and 22 cells (CAMSAP2 KO) from five independent experiments. (G) Average speed of spindle elongation for 5 minutes in anaphase shown in (F). n = 16 cells (WT) from six independent experiments and 20 cells (CAMSAP2 KO) from four independent experiments. Boxplots indicate 25th percentile, median, and 75th percentile values. **P < 0.01, Student’s t-test. (H) Temporal kinetics of the spindle elongation speed. Each gray dot corresponds to the value for each spindle. Black (WT) and red (CAMSAP2 KO) lines represent mean values of all the samples. The mean lines are selected to draw the graph at the bottom for simplicity. (G) and (H) share the dataset for quantification.

The absence of bridging fibers leads to impaired mitotic fidelity, resulting in chromosome segregation errors and mitotic delay [14,18]. As bridging fibers were reduced in CAMSAP2 KO cells (Fig 4E), we investigated whether CAMSAP2 KO affects mitotic fidelity. Chromosomes were visualized by Hoechst staining, and chromosomal behavior was monitored using live-cell imaging (Fig 5A).

We first recorded the timing of ‘first alignment’ when all the chromosomes first aligned to the equatorial plane, then the timing of ‘anaphase onset,’ and finally the timing of ‘completion of chromosome separation’ when two masses of sister chromatids were completely detached from each other during anaphase (Fig 5A). Duration from ‘first alignment’ to ‘anaphase onset’ was 24.98 ± 3.73 min for WT cells and 24.68 ± 3.17 min for CAMSAP2 KO cells, with no significant differences (Fig 5B). In contrast, the duration from ‘anaphase onset’ to ‘completion of chromosome separation’ was significantly longer in CAMSAP2 KO cells (Fig 5C). In WT cells, 80.77% of the mitotic cells segregated chromosomes properly (top row, Fig 5D), whereas 38.46% of CAMSAP2 KO cells were able to do the same. Chromosome segregation defects observed in CAMSAP2 KO cells included 11.5% of lagging chromosomes (red arrowheads) or 30.8% of chromatin bridge phenotype (yellow arrowheads). In addition, 19.2% of CAMSAP2 KO miotic cells failed to segregate chromosomes (bottom row). Spindle elongation during anaphase was measured using live-cell imaging with the microtubule-associated protein EB1-mCherry2 to visualize microtubules and spindle poles, and the time-lapse kinetics of spindle length relative to that of the first time point were recorded over time (defined as relative spindle length, Fig 5E). In WT cells, chromosomes were successfully segregated into two spindle poles, and the average speed of spindle elongation was 0.0207 ± 0.00142 μm/second, whereas that in CAMSAP2 KO cells was 0.0143 ± 0.00149 μm/second (Fig 5F and 5G). The speed of spindle elongation at each time point was calculated in WT and CAMSAP2 KO cells. The kinetics of anaphase spindle elongation can be divided into two phases: an earlier phase with higher speed and a later phase with slower speed, which may correspond to anaphase A and B, respectively (Fig 5F and 5H). The elongation speed was particularly low in the later phase, anaphase B (Fig 5F and 5H and S2 Table). These results suggest that the absence of CAMSAP2 hampers spindle elongation during anaphase, which may result in the failure of correct chromosome segregation.

## Discussion

Using mammalian epithelial Caco-2 cells, the present study revealed that the CAMSAP2 protein plays a role in mitosis, which has not been previously studied, as its localization was not detected in the mitotic spindle. These observations were confirmed by both anti-CAMSAP2 and EGFP-Camsap2 staining (Fig 1A; S1 and S3 Figs). Although we cannot exclude the possibility that a small number of CAMSAP2, too low to detect by our methods, might influence spindle dynamics, we conclude that CAMSAP2 is absent from the spindle during mitosis. This notion is supported by a systematic proteomic study detecting CAMSAPs as interphase-specific MAPs [41].

We also considered possibilities of partial reduction of other MAPs caused by CAMSAP2 KO. Although we cannot exclude the possibility that a partial reduction of CASMAP1 partially contributed the phenotype of CASMAP2 KO, CAMSAP1 localization to the spindle did not significantly change by CAMSAP2 KO (S7C and S7D Fig). Effects of CAMSAP2KO on protein levels of other MAPs have been systematically investigated in the previous study using rat Sertoli cells: CAMSAP2 KD did not affect protein levels of eight representative MAPs including CAMSAP1 and EB1 [48]. We particularly focused on EB1, as it reportedly binds to CAMSAP2 [49]. The effects of CASMAP2 KD on EB1 amounts on microtubules appear variable depending on cell types, as CAMSAP2 KD caused upregulation of EB1 on microtubules in HeLa cells [49], but downregulation in hepatocellular carcinoma [50], or no changes in rat Sertoli cells [48]. In our Caco-2 cells, CAMSAP2 KO caused no changes in fluorescence levels of EB1 proteins endogenously tagged with mcherry2 by chromosomal knock-in (Fig 5E). It is therefore unlikely that the CAMSAP2 KO phenotypes were due to reduction of other MAPs, although we cannot completely exclude the possibility.

Mitotic delay was observed in CAMSAP2-depleted cells, but not in CAMSAP3-depleted cells. In interphase cells, CAMSAP2 and CAMSAP3 perform redundant functions and stabilize the minus ends of non-centrosomal microtubules [20]. However, *in vitro* experiments showed that CAMSAP3 has a stronger affinity for microtubules than CAMSAP2 [21,22,24]. In CAMSAP2 KO cells, CAMSAP3 may stabilize microtubules more strongly than in WT cells, which may hamper microtubule reorganization upon mitotic entry.

We found that CAMSAP2 KO Caco-2 cells exhibited defects in spindle length and chromosome segregation, which resembled the phenotypes observed in the depletion of the *Drosophila* homolog, Patronin-depleted conditions [38,39]. Patronin localizes to the spindle poles where it binds to the minus end of spindle microtubules, protecting them from depolymerization by kinesin-13. However, CAMSAP2 was diffusely localized during mitosis and was not observed in the microtubules (Fig 1A). In addition, depletion of KIF2A, a mammalian homolog of kinesin-13, did not fully suppress the CAMSAP2 spindle phenotypes, particularly displaced spindle poles. Therefore, CAMSAP2 contributes to the organization of spindle microtubules in a manner distinct from that of Patronin.

CAMSAP2 in Caco-2 cells, as also observed in HeLa cells [21], was unphosphorylated during interphase and phosphorylated during mitosis. However, the relationship between phosphorylation and delocalization from spindle microtubules during mitosis remains unclear. Furthermore, whether similar mechanisms are employed to assemble mitotic spindles in other mammalian cells remains yet to be determined.

### Partial suppression of CAMSAP2 KO phenotypes by KIF2A depletion

KIF2A KD reduces depolymerization of centrosomal microtubules at spindle poles [51–53]. Although KIF2A appears to play similar roles to *Drosophila* Klp10A, the phenotype of KIF2A depletion is reportedly rather variable; KIF2A KD in general causes an increase in spindle length in HeLa, U2OS, and RPE-1 cells [44,54,55]; however, this phenotype was occasionally not observed [52]. In our study using Caco-2 cells, KIF2A KD did not significantly increase spindle length (Fig 3B). Although the reason for this remains unclear, we speculate that the contribution of KIF2A/Klp10A to spindle size varies depending on the organism or cell type. In general, centrosomal microtubules stabilize when KIF2A is depleted, resulting in the outward extension of the spindle. In Caco-2 cells, non-centrosomal bridging fibers may be predominant and are not subjected to depolymerization by KIF2A. Therefore, in KIF2-depleted Caco-2 cells, microtubules, including bridging fibers, may oppose the generation of an outward force to extend the spindle, thereby minimizing the increase in spindle size.

Both Patronin-depleted *Drosophila* cells and CAMSAP2 KO Caco-2 cells exhibited short spindles. Spindle length was restored by depleting the kinesin-13 proteins Klp10A and KIF2A [38] (Fig 3B). In *Drosophila*, the suppression by co-depletion may be explained by an antagonistic relationship at the spindle poles. In contrast, CAMSAP2 (and CAMSAP3) in Caco-2 cells did not show clear localization during mitosis. Therefore, this suppression may be due to distinct reasons. As bridging fibers were decreased by CAMSAP2 KO, spindle length may no longer be maintained upon microtubule stabilization in KIF2A-depleted cells, resulting in an increase in spindle size. However, the displaced centrosomes in CAMSAP2 KO cells were not suppressed by KIF2A co-depletion. This may be because a decrease in the number of bridging fibers, which causes the ‘displaced centrosomes’ phenotype in CAMSAP2 KO cells, cannot be overwhelmed by microtubule stabilization at the centrosomes by KIF2A depletion.

Therefore, although KIF2A appears to play similar roles to *Drosophila* Klp10A [44,54,55], it might differ between *Drosophila* and mammalian cells in terms of how the integrity of spindle poles is maintained by CAMSAP family proteins.

### How CAMSAP2 regulates spindle microtubules without clear localization

In CAMSAP2 KO mitotic cells, spindle intensity, particularly near the chromosomal region, decreased. This was mainly because of the reduction in bridging fibers, but not kinetochore microtubules (Fig 4A). Centrosomal microtubules become predominant in interphase CAMSAP2 depleted cells [20], and kinetochore microtubules contain centrosomal microtubules within the mitotic spindles. It is possible that these interphase centrosomal microtubules convert into mitotic spindles, thereby contributing to an increase in kinetochore microtubules.

How does CAMSAP2 affect mitotic spindle assembly, despite not showing a clear localization in mitotic spindles in WT cells? We speculated the following scenario regarding how CAMSAP2 affects the spindle during WT mitosis, as illustrated in a schematic (WT, S11A Fig).

In interphase, Caco-2 cells display non-centrosomal microtubules prevalent in the cytoplasm. Those non-centrosomal microtubules serve as a large pool of tubulin dimers even before mitotic onset, which can be used to rebuild the spindle upon entry into mitosis (WT, S11A Fig for the schematic). Most of CAMSAP-bearing microtubules displayed CAMSAP2 and CAMSAP3 simultaneously at the end (83.6%, CAMSAP2/3-MTs, S11B Fig). Besides, we found that a certain fraction of microtubules displayed CAMSAP2 only at the tip (12.8%, CAMSAP2-MTs), whereas a small fraction displayed CAMSAP3 only (3.5%, CAMSAP3-MTs). We therefore envision that such CAMSAP2-microtubules without CAMSAP3 might release tubulin dimers more efficiently than CAMSAP3-microtubules, as previously suggested by in vitro studies [21,22,24]. This might explain how CAMSAP2 contributes to efficient spindle assembly without localizing to the spindle. At a later stage, CAMSAP2 is delocalized from non-centrosomal microtubules, which assists further production of tubulin dimers.

As shown previously, CAMSAP2-depleted cells exhibit an increase in the number and length of centrosomal microtubules during interphase [20]. Therefore, CAMSAP2-depleted cells tend to maintain centrosomal microtubules, even upon mitotic entry (CAMSAP2 KO, S11 Fig). It is therefore possible that fewer free tubulin dimers are available for the reassembly of non-centrosomal microtubules in the CAMSAP2 KO spindle than in WT. This causes a reduction of Augmin in the CAMSAP2 KO spindle (Fig 4D), as Augmin uses non-centrosomal microtubules as its platform. As a result, microtubules in the CAMSAP2 KO spindle are not fully assembled compared to WT. However, careful analyses of microtubule reorganization during the G2/M transition are needed to confirm this hypothesis.

Augmin is implicated in the nucleation of kinetochore fibers [15] in addition to non-kinetochore microtubules such as bridging fibers [14]. Among the Augmin–γ-tubulin-mediated microtubules, for which CAMSAP2 is responsible, bridging fibers appears to mainly affect the chromosome segregation. This notion is based on the following results: (1) In cold treatment assays to selectively visualize kinetochore fibers, kinetochore microtubules tolerant to cold shock were largely comparable in WT and CAMSAP2 KO cells (Fig 4B and 4C). (2) More specifically, the intensities of the bridging and kinetochore fibers were quantified using super-resolution images (Fig 4E and 4F). In CAMSAP2 KO cells, although reduction in intensities was observed in both kinetochore and bridging fibers, the reduction was particularly evident with regard to bridging fibers (Fig 4F). From these results, we conclude that CAMSAP2 contributes mainly to bridging fibers and only slightly to kinetochore fibers.

Bridging fibers exert a pushing force and inter-kinetochore tension by directly pressing on kinetochore fibers and centrosomes, or by forming antiparallel overlapping microtubules and sliding them [14,18,56]. Because these forces contribute to chromosome alignment and segregation, CAMSAP2 KO cells can show displaced chromosomes from the metaphase plate and delayed chromosome segregation (Fig 5).

### How CAMSAP2- and CAMSAP3-KOs behaves differentially upon mitotic entry

In interphase, both CAMSAP2 and CAMSAP3 decorate the microtubule minus ends for stabilization; however, this study demonstrates that the mitotic phenotypes of CAMSAP2 KO and CAMSAP3 KO are distinct from each other. We hypothesized that this is because of their distinct affinities for microtubules. At mitotic onset, CAMSAP2 dissociates from microtubules, releasing a considerable amount of tubulin dimers into the cytoplasm. Furthermore, CAMSAP3 possesses a higher affinity towards microtubules than CAMSAP2 [21], implying that a smaller amount of tubulin dimers might be released by CAMSAP3.

Based on this hypothesis, interphase CAMSAP2 KO cells mostly show centrosomal microtubules (S11A Fig), resulting in a general shortage of free tubulin dimers that can be reassembled into non-centrosomal microtubules in the spindle. Furthermore, the remaining microtubules are mostly decorated with CAMSAP3 at the end, implying that additional free tubulin dimers are not sufficiently produced, assuming that CAMSAP3 does not efficiently release dimers. In contrast, CAMSAP3 KO cells can provide tubulin dimers, as remaining CAMSAP2-microtubules easily release dimers. Although CAMSAP proteins disperse when cells enter mitosis, preparation of tubulin dimers in interphase by the timing of mitotic entry might be a key for proper spindle assembly. These may cause a distinct phenotype; mitotic delay is observed in CAMSAP2 KO cells but not in CAMSAP3 KO cells.

Having explored the role of the CAMSAP2 protein in microtubule reorganization upon interphase–mitosis transition, further studies are required to understand how the minus ends of cytoplasmic microtubules are regulated as a preparation for upcoming mitotic entry, so that tubulin dimers can be efficiently reused at the proper time and geographically reassembled into distinct sites of the spindle composed of centrosomal as well as non-centrosomal microtubules.

## Supporting information

S1 Fig Endogenous localization of CAMSAP2 in interphase and mitosis. Endogenous localization of CAMSAP2 in each cell cycle stage of Caco-2 WT cells (A) and CAMSAP2 KO cells (B). Cells were fixed with methanol and stained for α-tubulin (green), CAMSAP2 (red) and DAPI (blue). The boxed regions have been enlarged, brightness-adjusted, and shown in the inset. Arrowheads, punctum localization of CAMSAP2 at microtubule ends. CAMSAP2 punctae seen in interphase WT cells were undetectable in CAMSAP2 KO cells (B). CAMSAP2 signals at spindle poles seen in WT mitosis (A) were concluded as non-specific signals, as similar signals were also detected in CAMSAP2 KO cells (B). Scale bars; 10 μm.
(TIF)

S2 Fig Endogenous localization of CAMSAP3 in interphase and mitosis. Endogenous localization of CAMSAP3 in each cell cycle stage of Caco-2 WT cells (A) and CAMSAP3 KO cells (B). Cells were fixed with methanol and stained for α-tubulin (green), CAMSAP3 (red) and DAPI (blue). The boxed region has been enlarged, brightness-adjusted and shown in the inset. CAMSAP3 punctae (arrowheads) was observed in interphase WT cells (A) but was undetectable in CAMSAP3 KO cells (B). Scale bars; 10 μm.
(TIF)

S3 Fig Localization of episomally expressed EGFP-tagged Camsap2 and Camsap3. Localization of EGFP-Camsap2 (A) and EGFP-Camsap3 (B) overexpressed from plasmids in interphase and mitosis (metaphase). Representative images acquired using an LSM980 Airyscan are shown. Cells were fixed with methanol and stained for GFP (green), β-tubulin (red) and DAPI (blue). Single z-planes corresponding to the boxed regions have been enlarged, brightness-adjusted and shown in insets. Arrowheads indicate EGFP-Camsap2 (A) and EGFP-Camsap3 (B) punctae at microtubule ends. (A) EGFP-Camsap2 punctae were undetectable in metaphase. (B) EGFP-Camsap3 signals were detected at spindle poles in mitosis when overexpressed. Scale bar; 10 μm.
(TIF)

S4 Fig Synchronization of cells by the STLC treatment. WT cells were synchronized to metaphase with 5μM STLC. Mitotic index with or without the STLC treatment was analyzed as in Fig 1D. n = 213 DMSO-treated cells (control) and n = 238 STLC-treated cells in an experiment. Mitotic index was 4.23% in asynchronous DMSO-treated cells, which was increased to 36.55% in STLC-treated cells.
(TIF)

S5 Fig Protein expression and cell cycle analyses in CAMSAP2 KO and CAMSAP3 KO cells. Depletion of CAMSAP2 in CAMSAP2 KO cells (A) and depletion of CAMSAP3 in CAMSAP3 KO cells (B) were confirmed by western blotting. GAPDH, loading control. MW, kDa. (C) FACS analyses for WT, CAMSAP2 KO and CAMSAP3 KO cells. Cells were stained with propidium iodide and analyzed using the cell analyzer Cytomics FC500MPL. No apparent peak differences were observed among the observed cells. Three independent experiments were conducted, and the representative data are shown.
(TIF)

S6 Fig Correlation assays between the spindle length and cell diameter. Correlation between the spindle length and cell diameter was tested in WT and CAMSAP2 KO cells. The dataset used in Fig 2A were shared with the test. Mad2-negative spindles were exclusively chosen as metaphase cells for quantification. Neither WT nor CAMSAP2 KO show correlation between the spindle length and cell diameter. n = 79 (WT) and 86 (CAMSAP2 KO) cells from 4 independent experiments. R, correlation coefficient.
(TIF)

S7 Fig CAMSAP2 KO phenotypes can be explained by the loss of CAMSAP2 per se. (A) Rescue experiments for CASMAP2 KO cells with episomal plasmids containing the EGFP-Camsap2 construct. Mad2-negative spindles were exclusively chosen as metaphase ones, and the spindle length and the degree of displaced centrosomes were quantified. The spindle length (left) was measured based on β-tubulin signals. n = 50 (WT + EGFP), 49 (CAMSAP2 KO + EGFP), and 41 (CAMSAP2 KO + EGFP-Camsap2) cells from 3 independent experiments. ****P < 0.0001, n.s.: P > 0.05, Welch’s t-test. Degree of displaced centrosomes (right) was calculated based on γ-tubulin signals at spindle poles as in Fig 2H. n = 55 (WT + EGFP), 52 (CAMSAP2 KO + EGFP) and 66 (CAMSAP2 KO + EGFP-Camsap2) cells from 3 independent experiments. ****P < 0.0001, Welch’s t-test. n.s.: P > 0.05, Welch’s t-test. Those indices for cells harboring the EGFP-Camsap2 plasmid were restored to the level comparable to WT + EGFP (control). (B) Western blotting for CAMSAP1. The band intensity of CAMSAP1 was normalized with the GAPDH (control) intensity. MW (kDa) is shown in the left. Mean ± SD from 3 independent experiments. n.s.: P > 0.05, Student’s t-test. (C) Localization of 3×GFP-CAMSAP1 (red, three tandem copies of GFP are fused with CAMSAP1 at the N-terminus) to the metaphase spindle in WT and CAMSAP2 KO cells. Microtubules were stained with SiR-tubulin (green) and DNA with Hoechst (blue). CAMSAP1 signals were detected on spindle microtubules both in WT and CAMSAP2 KO cells. Scale bar; 10 μm. (D) Endogenous localization of CAMSAP1 to the spindle. Cells were fixed with methanol and stained for α-tubulin (green), CAMSAP1 (red) and DAPI (blue). CAMSAP1 signals were detected at spindle poles and microtubules in WT and CAMSAP2 KO cells. The background intensity was subtracted to calculate the CAMSAP1 intensity on the spindle. No significant differences in the amount of CAMSAP1 were seen between WT and CAMSAP2 KO cells. n = 81 (WT) and 61 (CAMSAP2 KO) cells, 3 independent experiments. n.s.: P > 0.05, Student’s t-test. Scale bars; 10 μm. (E) Western blotting for CAMSAP3 in CAMSAP 2KO cells. The band intensity of CAMSAP3 was normalized with the GAPDH (control) intensity. MW (kDa) is shown in the left. Mean ± SD from 3 independent experiments. n.s.: P > 0.05, Student’s t-test.
(TIF)

S8 Fig CAMSAP2 localization in interphase and mitotic CAMSAP3 KO cells. Endogenous localization of CAMSAP2 in interphase and each mitotic phase in CAMSAP3 KO cells. Methanol-fixed cells were stained for α-tubulin (green), CAMSAP2 (red) and DAPI (blue). The boxed region has been enlarged, brightness-adjusted and shown in the inset. Arrowheads, stretched CAMSAP2 signals at the microtubule ends, as previously reported [20]. CAMSAP2 signals were undetectable in mitotic cells. CAMSAP2 signals at spindle poles were concluded as non-specific ones as were also detected in CAMSAP2 KO cells (see S1B Fig). Scale bars; 10 μm.
(TIF)

S9 Fig Depletion of KIF2A by siRNA. Depletion of KIF2A was confirmed by western blotting in WT, CAMSAP2 KO and CAMSAP3 KO cells with (si KIF2A) or without (si Control) KIF2A knockdown. GAPDH, the loading control. MW (kDa) is shown in the left.
(TIF)

S10 Fig Mitotic phenotypes caused by co-depletion of CAMSAP2 and HAUS6. (A,B) Western blotting for γ-tubulin (A) and HAUS6 (B) in WT and CAMSAP2 KO cells. The band intensities were normalized with the GAPDH (control) intensity. MW (kDa) is shown in the left. Mean ± SD from 3 independent experiments. n.s.: P > 0.05, Student’s t-test. (C) Depletion of HAUS6 by siRNA was confirmed by western blotting. GAPDH, loading control. (D) The spindle length in HAUS6/control-depletion in WT and CAMSAP2 KO cells. Cells were fixed with 2% PFA and stained for α-tubulin (green), BubR1 (red) and DNA (blue). Representative images acquired by an LSM980 Airyscan are shown. BubR1-negative spindles were exclusively chosen as metaphase ones to measure the metaphase spindle length based on α-tubulin signals. n = 55 cells (si control WT), 58 (si HAUS6 WT), 47 (si control CAMSAP2 KO) and 66 (si HAUS6 CAMSAP2 KO) from 3 independent experiments. Boxplots (left) indicate 25th percentile, median and 75th percentile. ****P < 0.0001, **P < 0.01 Student’s t-test. The percentage decrease of spindle length (right) in WT and CAMSAP2 KO cells upon HASU6 co-depletion normalized by control cells. HAUS6 depletion reduced the spindle size to a similar extent in WT and in CAMSAP2 KO cells, although the effect appeared slightly more in the CAMSAP2 KO despite of no statistical significance. This indicates that the effect of CAMSAP2 in spindle length determination is largely through the Augmin pathway. Mean ± SEM of 3 independent experiments. n.s.: P > 0.05, Student’s t-test. Scale bar; 10 μm. (E) The γ-tubulin intensity in the spindle excluding spindle poles. Cells were fixed with 2% PFA and stained for α-tubulin (green), γ-tubulin (red) and DAPI (blue). The background intensity off the spindle was subtracted to calculate the γ-tubulin intensity on the spindle. The significant reduction in the amount of γ-tubulin intensity in si control WT and si control CAMSAP2 KO was no longer seen si HAUS6 WT and si HAUS6 CAMSAP2 KO. n = 51 cells (si control WT), 44 (si HAUS6 WT), 50 (si control CAMSAP2 KO), and 47 (si HAUS6 CAMSAP2 KO) from 3 independent experiments. Boxplots indicate 25th percentile, median and 75th percentile values. ****P < 0.0001, Welch’s t-test, n.s. > 0.05, Student’s t-test. Scale bar; 10 μm. (F) Organization of bridging fibers in single and double depletion. Representative images of a single z-plane acquired by an LSM980 Airyscan are shown. WT and CAMSAP2 KO cells with (si HAUS6) or without (si Control) HAUS6 knockdown were fixed with 2% PFA and stained for α-tubulin (green), CREST (red) and DAPI (blue). Boxed regions including representative sister kinetochore fibers have been magnified, brightness-adjusted and shown in insets. Arrowheads indicate a bridging fiber. Schematics are shown below each image. Frequencies (%) of sister kinetochore fibers that accompany bridging fibers behind are shown (bottom). The significant reduction was seen in bridging fibers in si control WT and si control CAMSAP2 KO, which was no longer seen in si HAUS6 WT and si HAUS6 CAMSAP2 KO. n = 28 pairs of kinetochores from 14 cells (si control WT), 38 from 20 cells (si control CAMSAP2 KO), 35 from 14 cells (si HAUS6 WT), and 40 from 18 cells (si HAUS6 CAMSAP2 KO) from 3 independent experiments. Mean ± SD of 3 independent experiments. *P < 0.05, Welch’s t-test. n.s.: P > 0.05, Student’s t-test. Scale bar; 10 μm.
(TIF)

S11 Fig A hypothetic model for spindle assembly in WT and CAMSAP KO cells. (A) Schematics for hypotheses: How CAMSAP2 contributes to spindle assembly upon entry into mitosis. See text for details. (B) Simultaneous observation of CAMSAP2 and CAMSAP3 localization in Caco-2 cells. Either CAMSAP2-GFP (top) or CAMSAP3-GFP (bottom) was ectopically expressed in wild-type cells. Cells were fixed with methanol and stained with anti-GFP (green), α-tubulin (gray), and DAPI (blue). CAMSAP3 (top) or CAMSAP2 (bottom) was stained with anti-CAMSAP3 or anti-CAMSAP2 antibody, respectively. Boxed regions are enlarged to visualize CAMSAP2 and CAMSAP3 punctae at microtubule ends. Microtubule ends with CAMSAP2 only (yellow arrowheads) or CAMSAP3 only (blue arrowheads) are indicated. Scale bars; 10 μm. The chart shows percentages of microtubules decorated with either or both CAMSAP2 and CAMSAP3 in interphase (N = 3 cells, n = 102, 97, 118 microtubules for +pEGFP-CAMSAP2; n = 74, 118, 78 for +pEGFP-CAMSAP3. Data are presented as mean ± standard error of the mean (SEM). ****P < 0.0001, ***P < 0.001, **P < 0.01, *P < 0.05, One-way ANOVA followed by Tukey’s multiple comparison tests.
(TIF)

S1 Table Primers used to construct EB1-mCherry2 cell lines. (XLSX)

S2 Table Kinetics of spindle elongation speed over time during anaphase. The data used for drawing Fig 5H. Spindle elongation speed was calculated every 20 seconds for 5 minutes in anaphase (μm/sec). Values are shown as averages of observed cells (n = 16 [WT] and 20 [CAMSAP2 KO] cells at each timepoint). asterisks = P < 0.05, Student’s t-test.
(XLSX)

S1 Raw images Raw images originally obtained in Western blotting assays shown in Figs 1B, S5, S7, S9 and S10. (PDF)

S2 Raw images (PDF)

S1 File (DOCX)
