# Supplementary figures and images for: CAMSAP2 is required for bridging fiber assembly to ensure mitotic spindle assembly and chromosome segregation in human epithelial Caco-2 cells
Source: PLoS One. 2025 Jan 9;20(1):e0308150. doi: 10.1371/journal.pone.0308150 (PMC11717264; doi:10.1371/journal.pone.0308150)

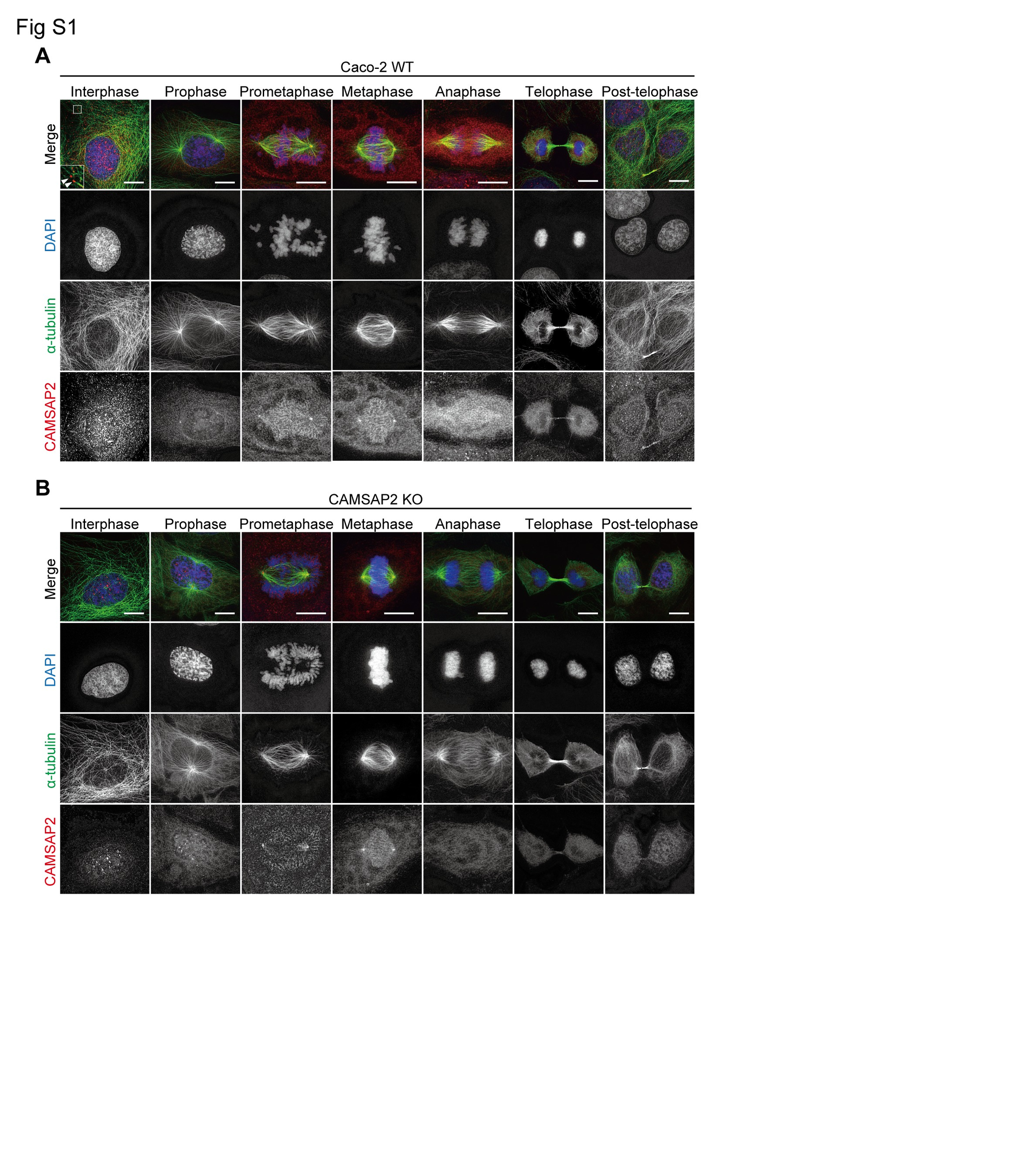

Supplement: S1 Fig — Endogenous localization of CAMSAP2 in each cell cycle stage of Caco-2 WT cells (A) and CAMSAP2 KO cells (B). Cells were fixed with methanol and stained for α-tubulin (green), CAMSAP2 (red) and DAPI (blue). The boxed regions have been enlarged, brightness-adjusted, and shown in the inset. Arrowheads, punctum localization of CAMSAP2 at microtubule ends. CAMSAP2 punctae seen in interphase WT cells were undetectable in CAMSAP2 KO cells (B). CAMSAP2 signals at spindle poles seen in WT mitosis (A) were concluded as non-specific signals, as similar signals were also detected in CAMSAP2 KO cells (B). Scale bars; 10 μm. (TIF) [file pone.0308150.s001.tif]

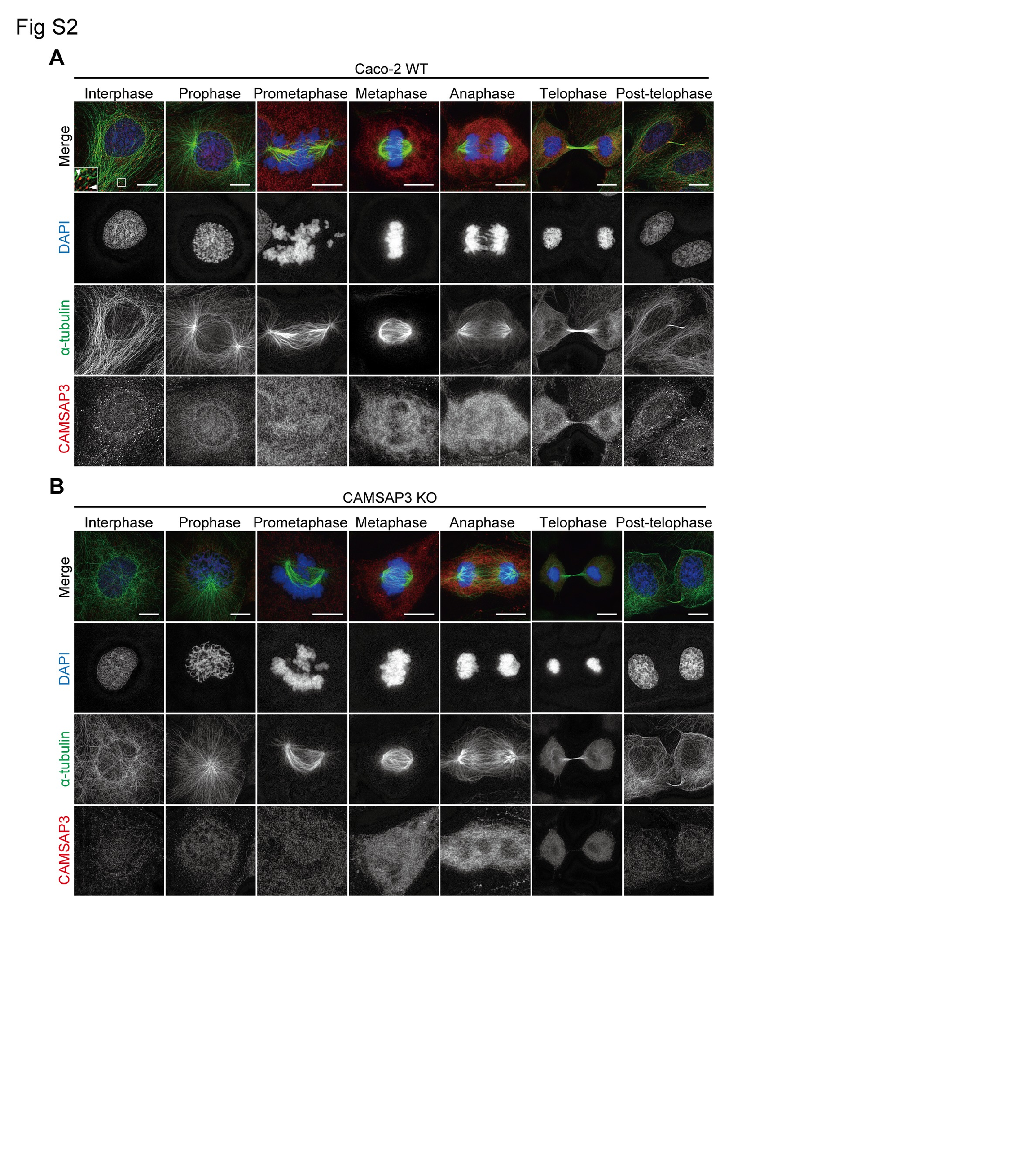

Supplement: S2 Fig — Endogenous localization of CAMSAP3 in each cell cycle stage of Caco-2 WT cells (A) and CAMSAP3 KO cells (B). Cells were fixed with methanol and stained for α-tubulin (green), CAMSAP3 (red) and DAPI (blue). The boxed region has been enlarged, brightness-adjusted and shown in the inset. CAMSAP3 punctae (arrowheads) was observed in interphase WT cells (A) but was undetectable in CAMSAP3 KO cells (B). Scale bars; 10 μm. (TIF) [file pone.0308150.s002.tif]

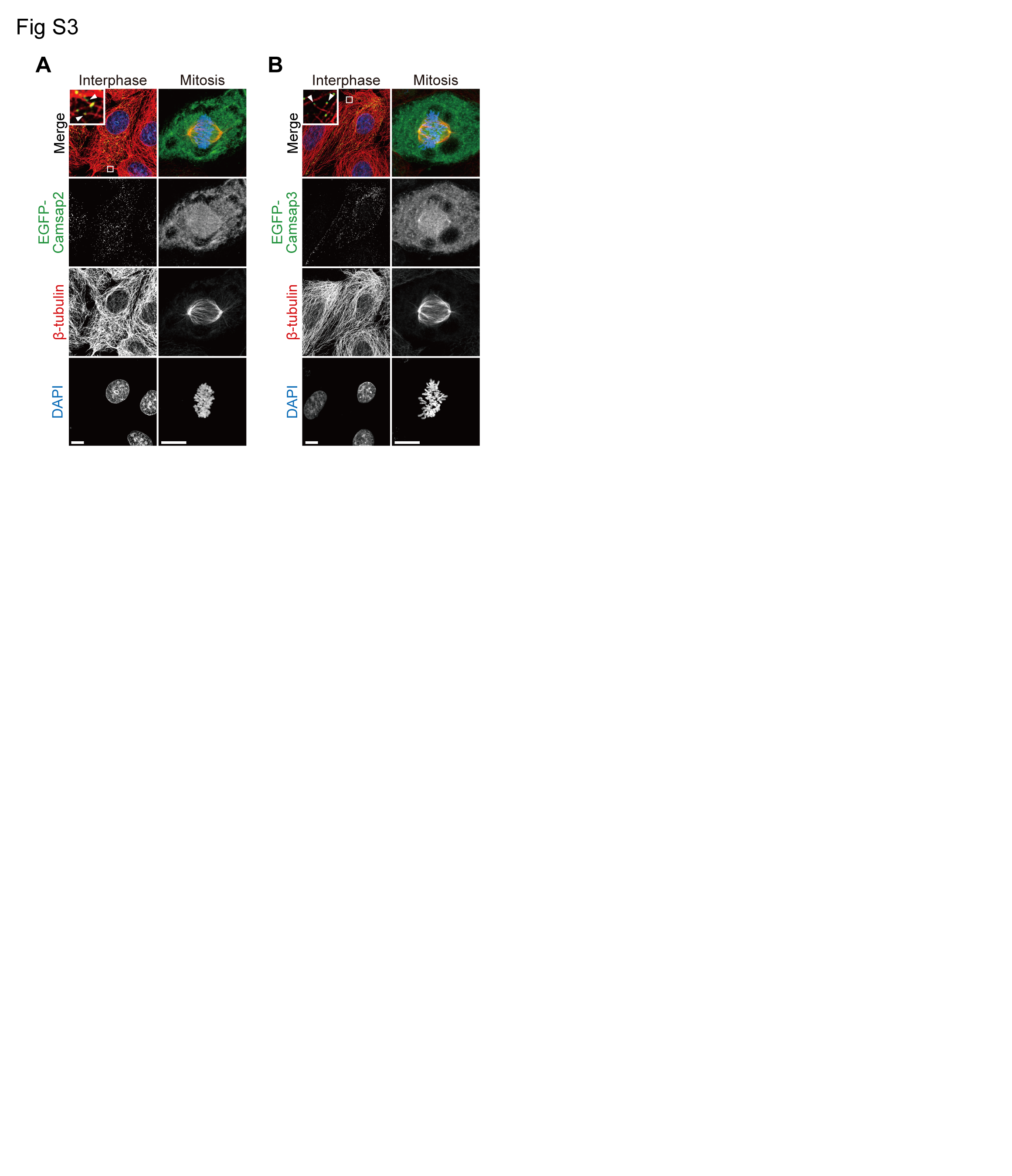

Supplement: S3 Fig — Localization of EGFP-Camsap2 (A) and EGFP-Camsap3 (B) overexpressed from plasmids in interphase and mitosis (metaphase). Representative images acquired using an LSM980 Airyscan are shown. Cells were fixed with methanol and stained for GFP (green), β-tubulin (red) and DAPI (blue). Single z-planes corresponding to the boxed regions have been enlarged, brightness-adjusted and shown in insets. Arrowheads indicate EGFP-Camsap2 (A) and EGFP-Camsap3 (B) punctae at microtubule ends. (A) EGFP-Camsap2 punctae were undetectable in metaphase. (B) EGFP-Camsap3 signals were detected at spindle poles in mitosis when overexpressed. Scale bar; 10 μm. (TIF) [file pone.0308150.s003.tif]

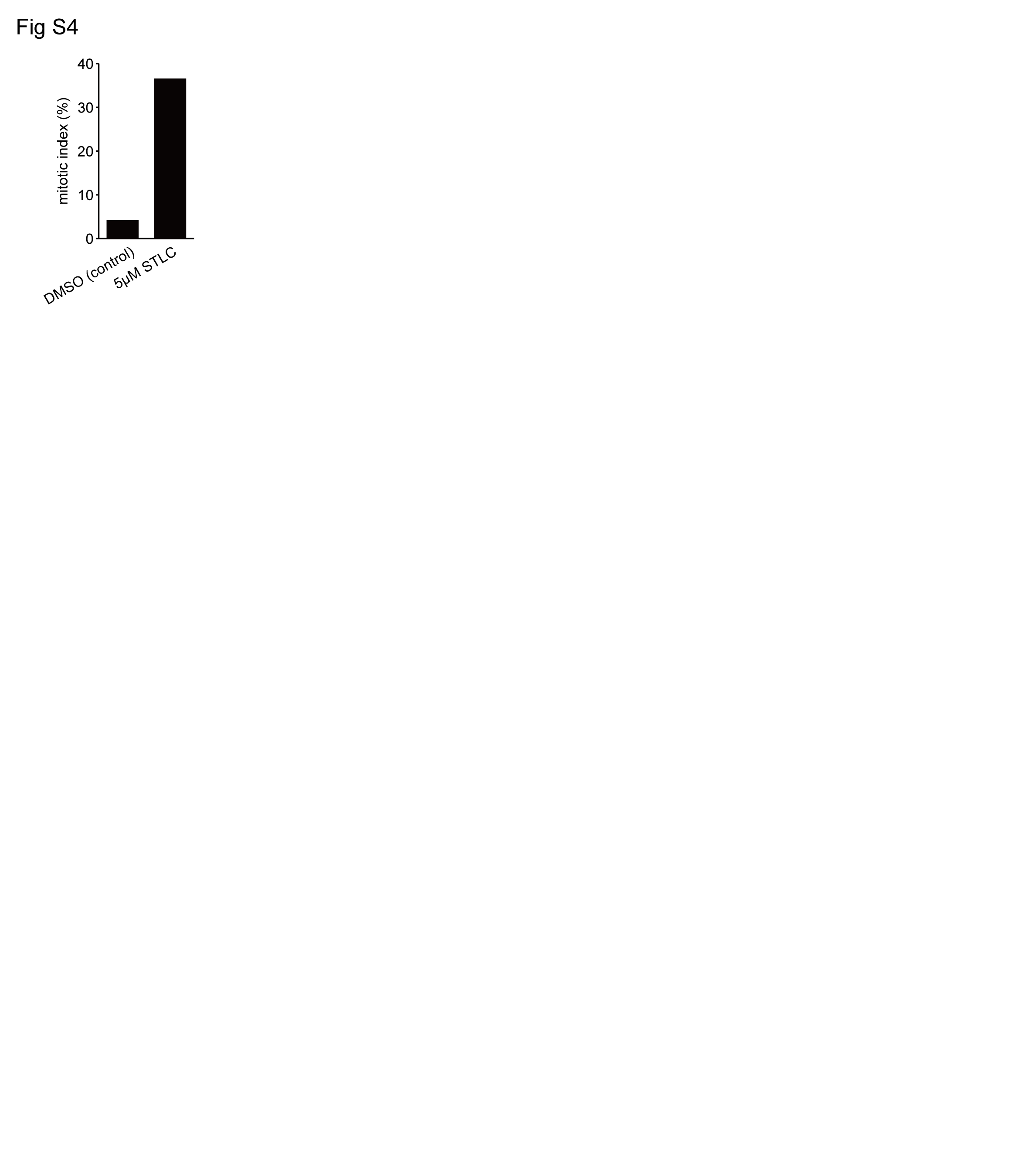

Supplement: S4 Fig — WT cells were synchronized to metaphase with 5μM STLC. Mitotic index with or without the STLC treatment was analyzed as in Fig 1D. n = 213 DMSO-treated cells (control) and n = 238 STLC-treated cells in an experiment. Mitotic index was 4.23% in asynchronous DMSO-treated cells, which was increased to 36.55% in STLC-treated cells. (TIF) [file pone.0308150.s004.tif]

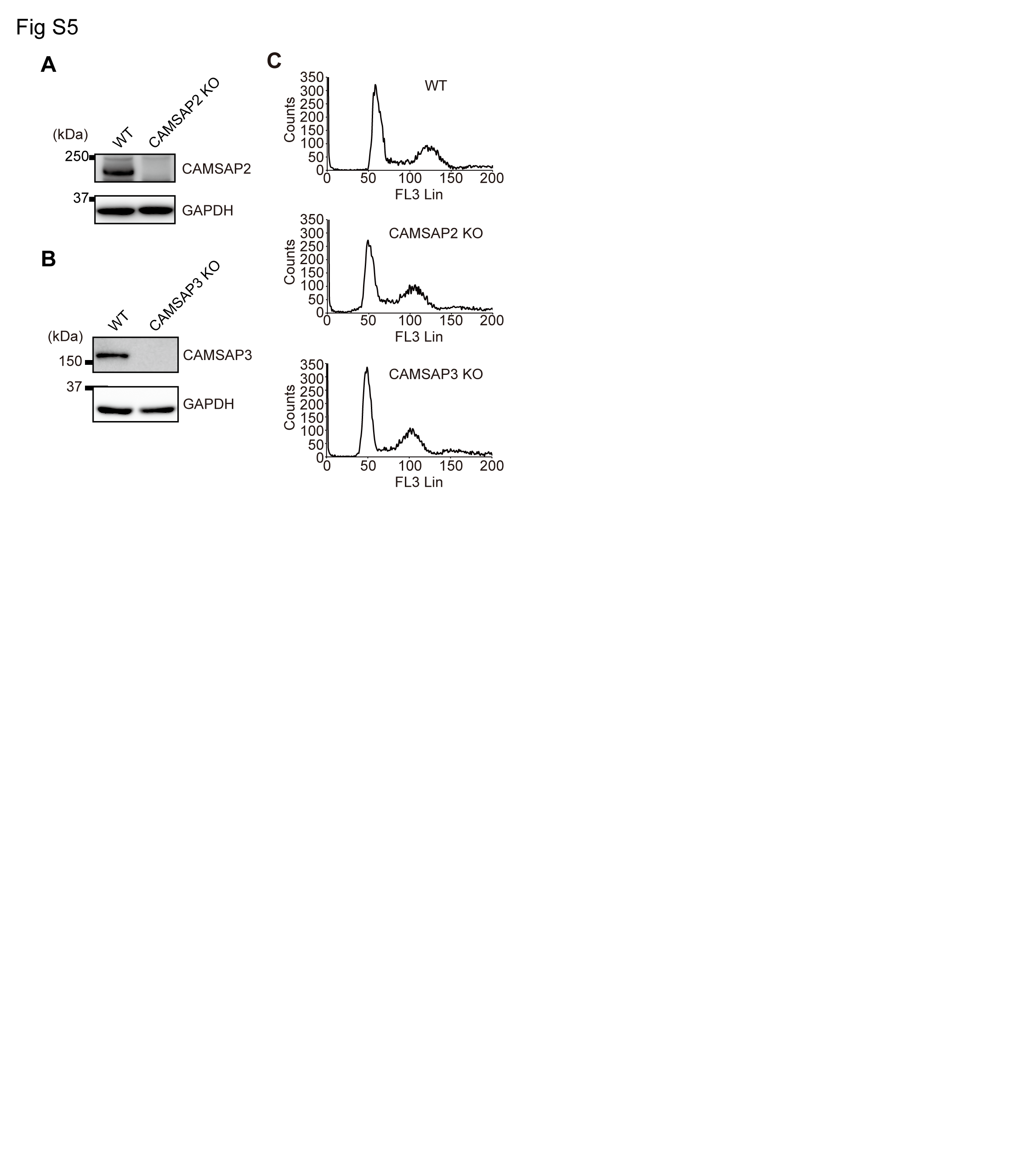

Supplement: S5 Fig — Depletion of CAMSAP2 in CAMSAP2 KO cells (A) and depletion of CAMSAP3 in CAMSAP3 KO cells (B) were confirmed by western blotting. GAPDH, loading control. MW, kDa. (C) FACS analyses for WT, CAMSAP2 KO and CAMSAP3 KO cells. Cells were stained with propidium iodide and analyzed using the cell analyzer Cytomics FC500MPL. No apparent peak differences were observed among the observed cells. Three independent experiments were conducted, and the representative data are shown. (TIF) [file pone.0308150.s005.tif]

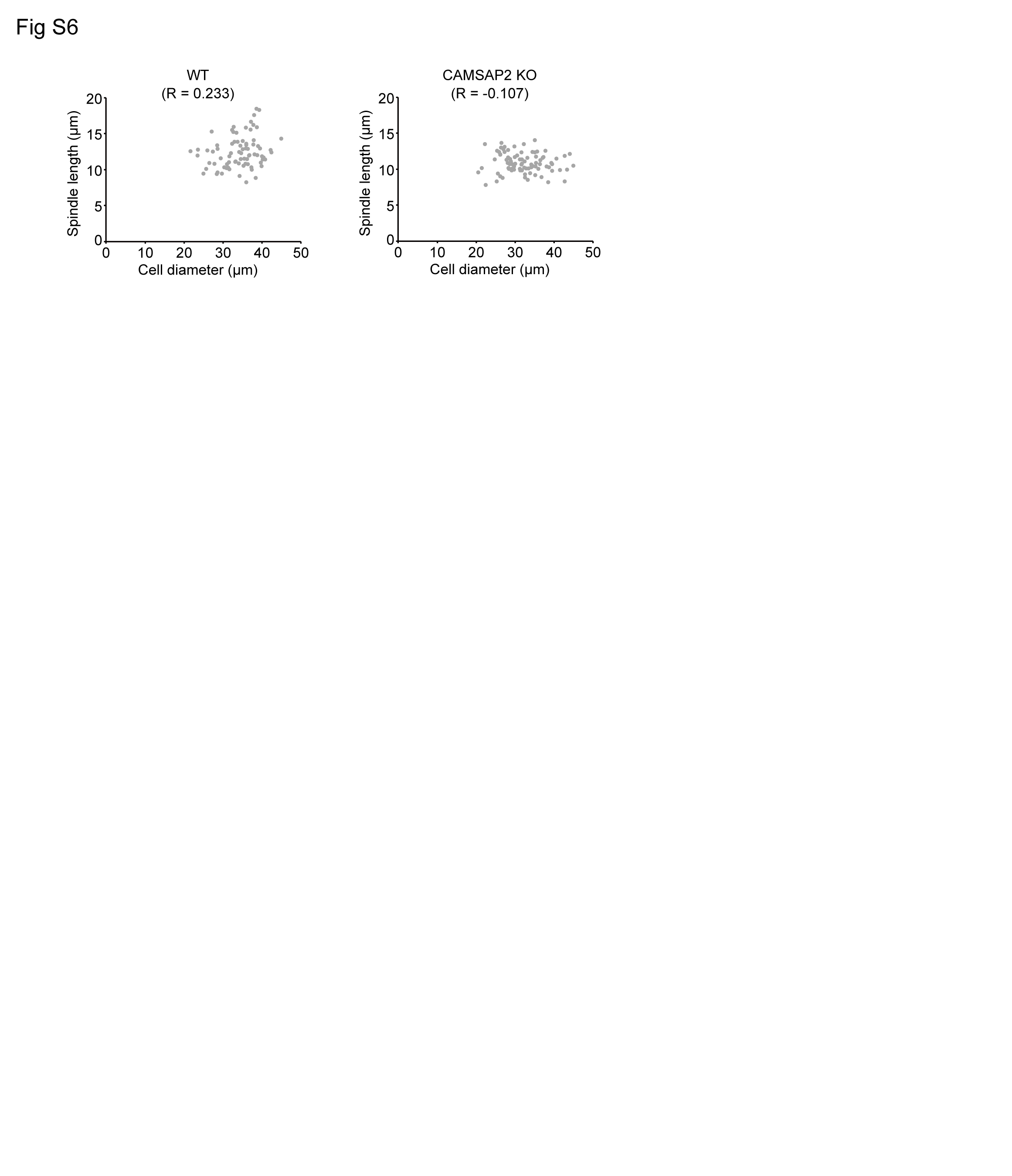

Supplement: S6 Fig — Correlation between the spindle length and cell diameter was tested in WT and CAMSAP2 KO cells. The dataset used in Fig 2A were shared with the test. Mad2-negative spindles were exclusively chosen as metaphase cells for quantification. Neither WT nor CAMSAP2 KO show correlation between the spindle length and cell diameter. n = 79 (WT) and 86 (CAMSAP2 KO) cells from 4 independent experiments. R, correlation coefficient. (TIF) [file pone.0308150.s006.tif]

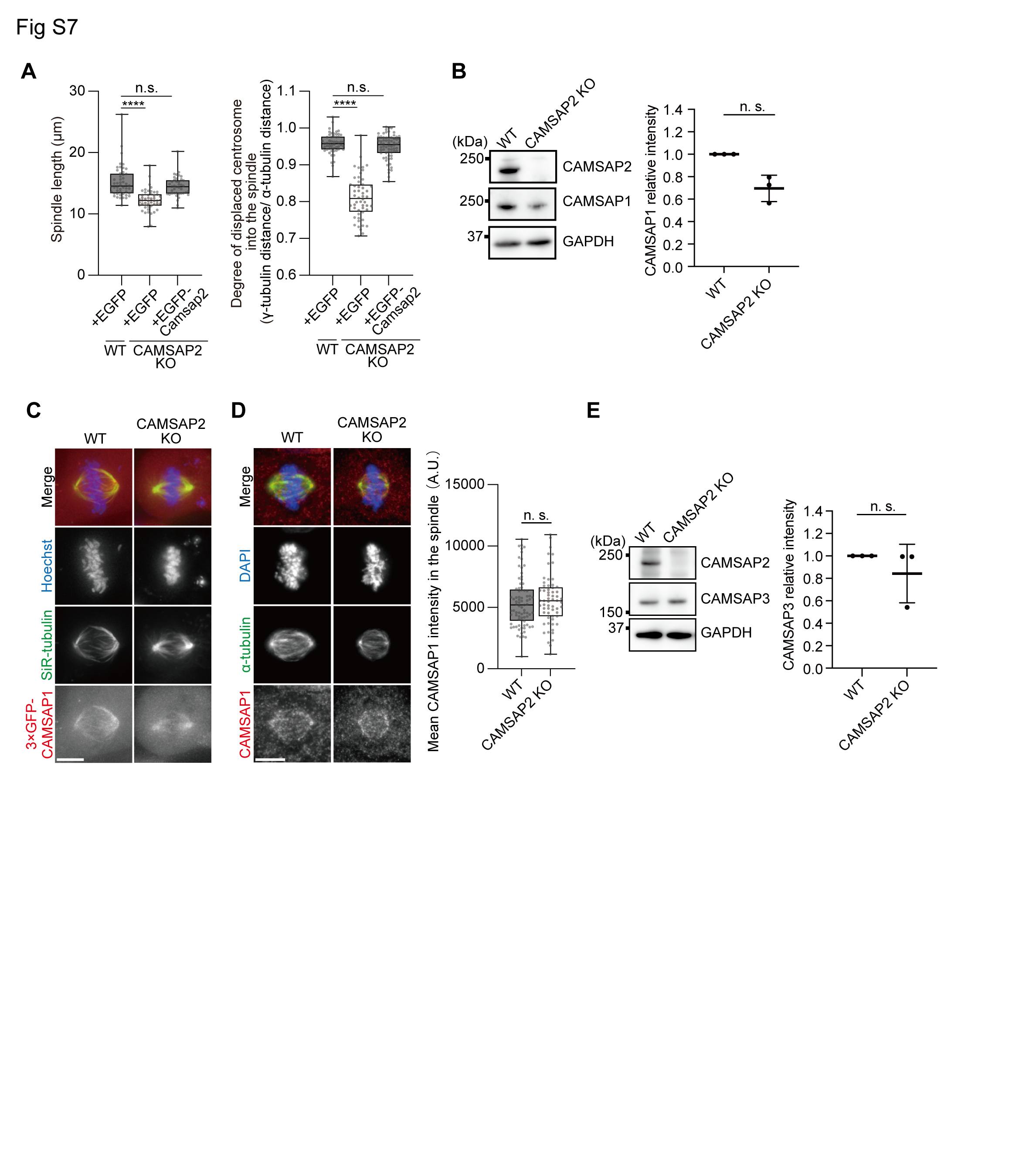

Supplement: S7 Fig — (A) Rescue experiments for CASMAP2 KO cells with episomal plasmids containing the EGFP-Camsap2 construct. Mad2-negative spindles were exclusively chosen as metaphase ones, and the spindle length and the degree of displaced centrosomes were quantified. The spindle length (left) was measured based on β-tubulin signals. n = 50 (WT + EGFP), 49 (CAMSAP2 KO + EGFP), and 41 (CAMSAP2 KO + EGFP-Camsap2) cells from 3 independent experiments. ****P < 0.0001, n.s.: P > 0.05, Welch’s t-test. Degree of displaced centrosomes (right) was calculated based on γ-tubulin signals at spindle poles as in Fig 2H. n = 55 (WT + EGFP), 52 (CAMSAP2 KO + EGFP) and 66 (CAMSAP2 KO + EGFP-Camsap2) cells from 3 independent experiments. ****P < 0.0001, Welch’s t-test. n.s.: P > 0.05, Welch’s t-test. Those indices for cells harboring the EGFP-Camsap2 plasmid were restored to the level comparable to WT + EGFP (control). (B) Western blotting for CAMSAP1. The band intensity of CAMSAP1 was normalized with the GAPDH (control) intensity. MW (kDa) is shown in the left. Mean ± SD from 3 independent experiments. n.s.: P > 0.05, Student’s t-test. (C) Localization of 3×GFP-CAMSAP1 (red, three tandem copies of GFP are fused with CAMSAP1 at the N-terminus) to the metaphase spindle in WT and CAMSAP2 KO cells. Microtubules were stained with SiR-tubulin (green) and DNA with Hoechst (blue). CAMSAP1 signals were detected on spindle microtubules both in WT and CAMSAP2 KO cells. Scale bar; 10 μm. (D) Endogenous localization of CAMSAP1 to the spindle. Cells were fixed with methanol and stained for α-tubulin (green), CAMSAP1 (red) and DAPI (blue). CAMSAP1 signals were detected at spindle poles and microtubules in WT and CAMSAP2 KO cells. The background intensity was subtracted to calculate the CAMSAP1 intensity on the spindle. No significant differences in the amount of CAMSAP1 were seen between WT and CAMSAP2 KO cells. n = 81 (WT) and 61 (CAMSAP2 KO) cells, 3 independent experiments. n.s.: P > 0.05, Student’s t-test. Sca [file pone.0308150.s007.tif]

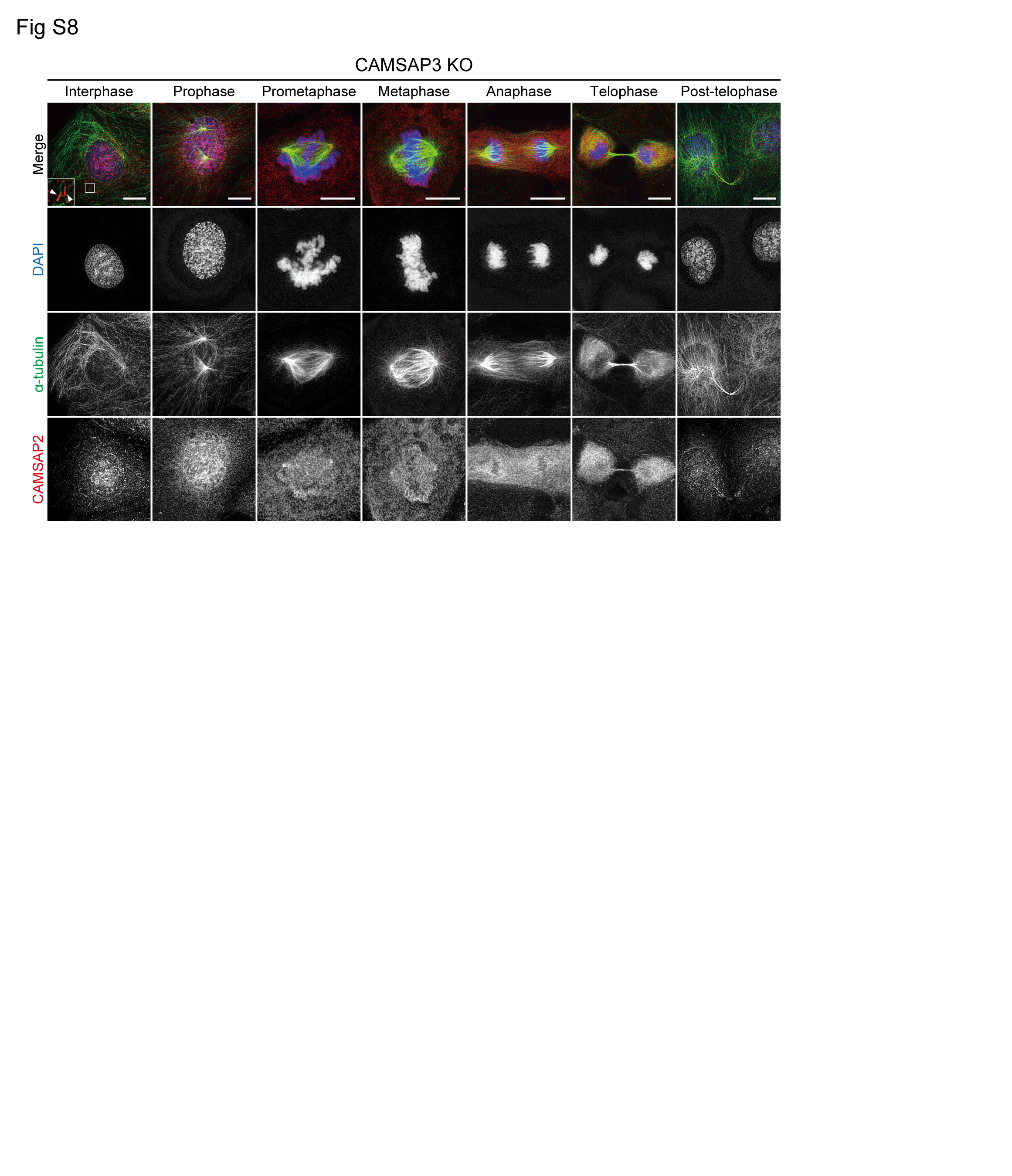

Supplement: S8 Fig — Endogenous localization of CAMSAP2 in interphase and each mitotic phase in CAMSAP3 KO cells. Methanol-fixed cells were stained for α-tubulin (green), CAMSAP2 (red) and DAPI (blue). The boxed region has been enlarged, brightness-adjusted and shown in the inset. Arrowheads, stretched CAMSAP2 signals at the microtubule ends, as previously reported [20]. CAMSAP2 signals were undetectable in mitotic cells. CAMSAP2 signals at spindle poles were concluded as non-specific ones as were also detected in CAMSAP2 KO cells (see S1B Fig). Scale bars; 10 μm. (TIF) [file pone.0308150.s008.tif]

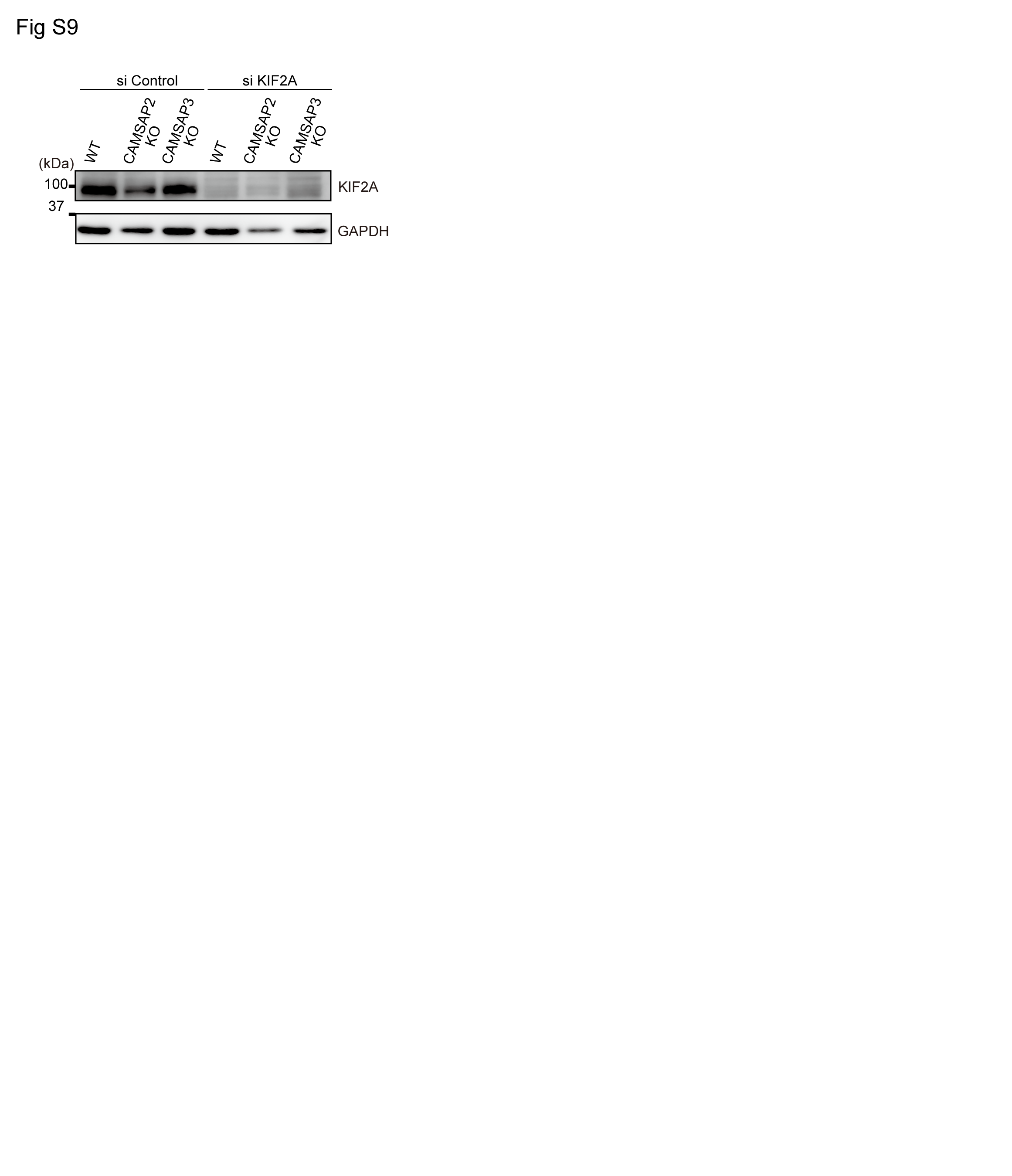

Supplement: S9 Fig — Depletion of KIF2A was confirmed by western blotting in WT, CAMSAP2 KO and CAMSAP3 KO cells with (si KIF2A) or without (si Control) KIF2A knockdown. GAPDH, the loading control. MW (kDa) is shown in the left. (TIF) [file pone.0308150.s009.tif]

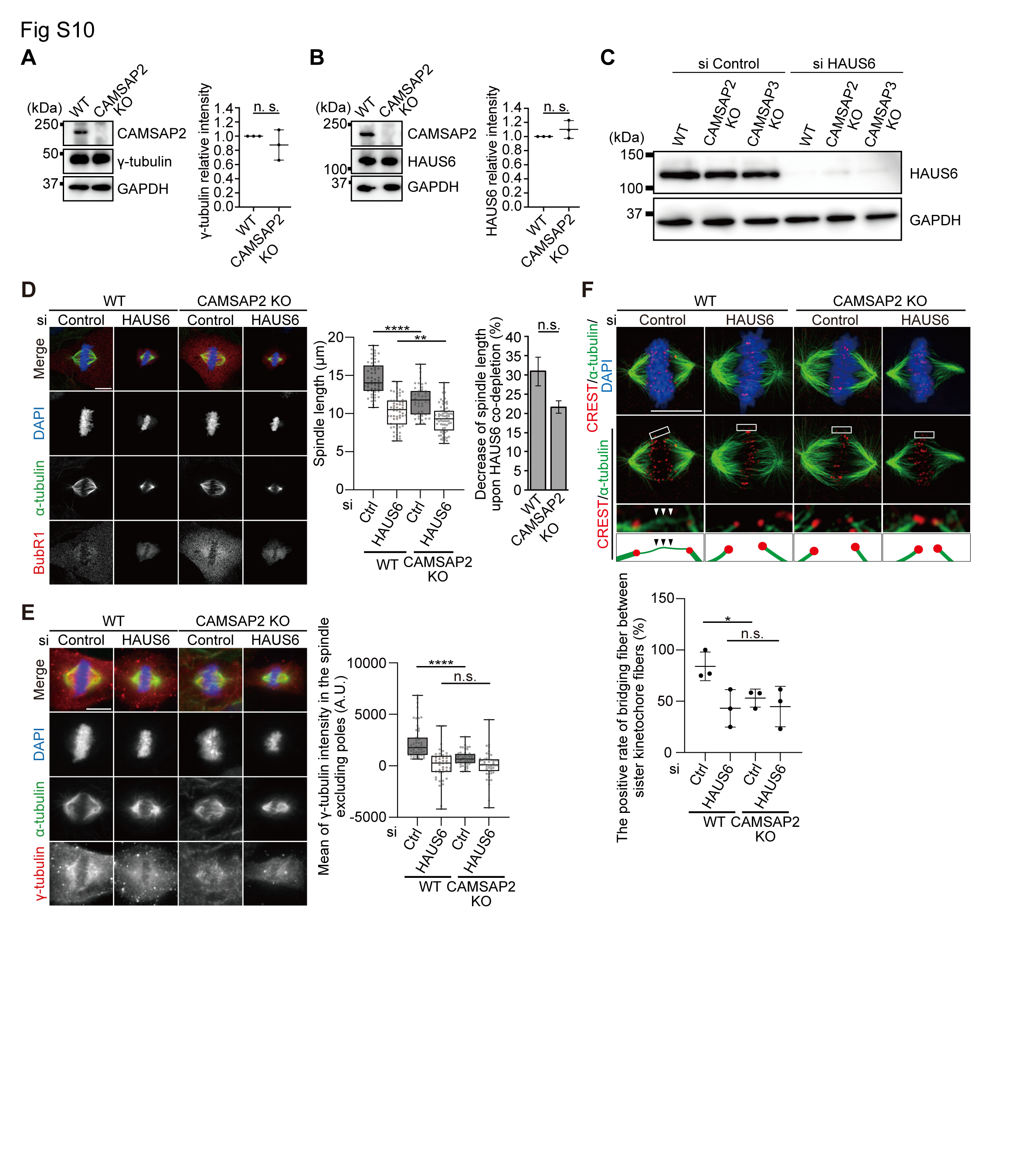

Supplement: S10 Fig — (A,B) Western blotting for γ-tubulin (A) and HAUS6 (B) in WT and CAMSAP2 KO cells. The band intensities were normalized with the GAPDH (control) intensity. MW (kDa) is shown in the left. Mean ± SD from 3 independent experiments. n.s.: P > 0.05, Student’s t-test. (C) Depletion of HAUS6 by siRNA was confirmed by western blotting. GAPDH, loading control. (D) The spindle length in HAUS6/control-depletion in WT and CAMSAP2 KO cells. Cells were fixed with 2% PFA and stained for α-tubulin (green), BubR1 (red) and DNA (blue). Representative images acquired by an LSM980 Airyscan are shown. BubR1-negative spindles were exclusively chosen as metaphase ones to measure the metaphase spindle length based on α-tubulin signals. n = 55 cells (si control WT), 58 (si HAUS6 WT), 47 (si control CAMSAP2 KO) and 66 (si HAUS6 CAMSAP2 KO) from 3 independent experiments. Boxplots (left) indicate 25th percentile, median and 75th percentile. ****P < 0.0001, **P < 0.01 Student’s t-test. The percentage decrease of spindle length (right) in WT and CAMSAP2 KO cells upon HASU6 co-depletion normalized by control cells. HAUS6 depletion reduced the spindle size to a similar extent in WT and in CAMSAP2 KO cells, although the effect appeared slightly more in the CAMSAP2 KO despite of no statistical significance. This indicates that the effect of CAMSAP2 in spindle length determination is largely through the Augmin pathway. Mean ± SEM of 3 independent experiments. n.s.: P > 0.05, Student’s t-test. Scale bar; 10 μm. (E) The γ-tubulin intensity in the spindle excluding spindle poles. Cells were fixed with 2% PFA and stained for α-tubulin (green), γ-tubulin (red) and DAPI (blue). The background intensity off the spindle was subtracted to calculate the γ-tubulin intensity on the spindle. The significant reduction in the amount of γ-tubulin intensity in si control WT and si control CAMSAP2 KO was no longer seen si HAUS6 WT and si HAUS6 CAMSAP2 KO. n = 51 cells (si control WT), 44 (si HAUS6 WT), 50 (si control [file pone.0308150.s010.tif]

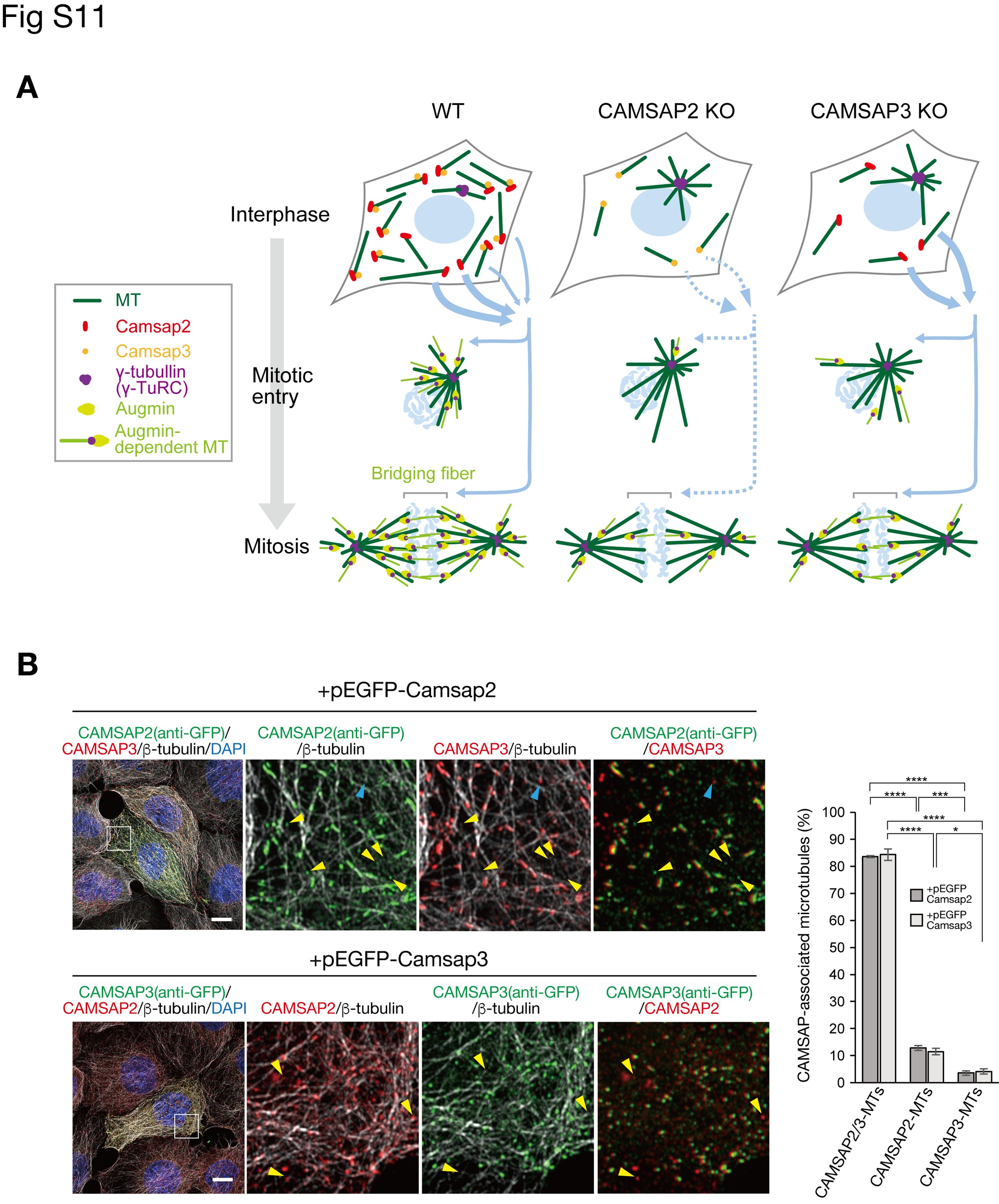

Supplement: S11 Fig — (A) Schematics for hypotheses: How CAMSAP2 contributes to spindle assembly upon entry into mitosis. See text for details. (B) Simultaneous observation of CAMSAP2 and CAMSAP3 localization in Caco-2 cells. Either CAMSAP2-GFP (top) or CAMSAP3-GFP (bottom) was ectopically expressed in wild-type cells. Cells were fixed with methanol and stained with anti-GFP (green), α-tubulin (gray), and DAPI (blue). CAMSAP3 (top) or CAMSAP2 (bottom) was stained with anti-CAMSAP3 or anti-CAMSAP2 antibody, respectively. Boxed regions are enlarged to visualize CAMSAP2 and CAMSAP3 punctae at microtubule ends. Microtubule ends with CAMSAP2 only (yellow arrowheads) or CAMSAP3 only (blue arrowheads) are indicated. Scale bars; 10 μm. The chart shows percentages of microtubules decorated with either or both CAMSAP2 and CAMSAP3 in interphase (N = 3 cells, n = 102, 97, 118 microtubules for +pEGFP-CAMSAP2; n = 74, 118, 78 for +pEGFP-CAMSAP3. Data are presented as mean ± standard error of the mean (SEM). ****P < 0.0001, ***P < 0.001, **P < 0.01, *P < 0.05, One-way ANOVA followed by Tukey’s multiple comparison tests. (TIF) [file pone.0308150.s011.tif]
