# Supplementary material for: CAMSAP2 is required for bridging fiber assembly to ensure mitotic spindle assembly and chromosome segregation in human epithelial Caco-2 cells
Source: PLoS One. 2025 Jan 9;20(1):e0308150. doi: 10.1371/journal.pone.0308150 (PMC11717264; doi:10.1371/journal.pone.0308150)

Figure 1B (Upper)

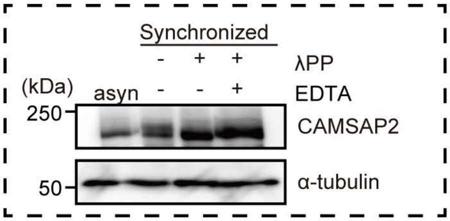

< Original images of blots >

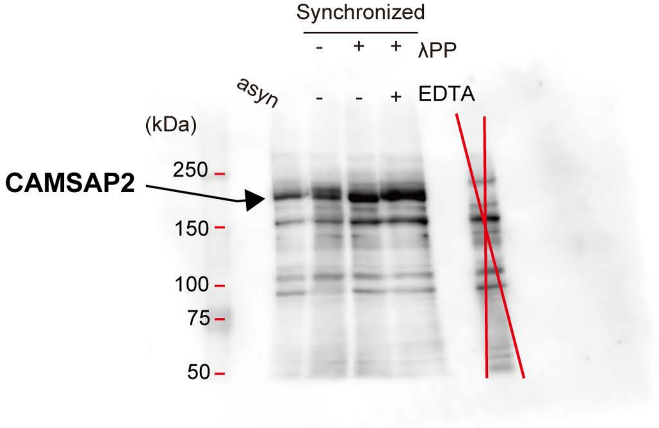

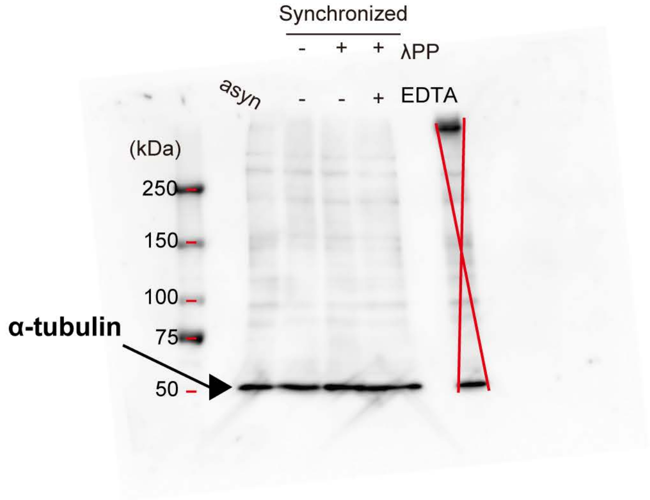

Figure 1B (Lower)

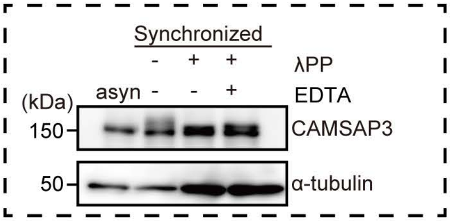

< Original images of blots >

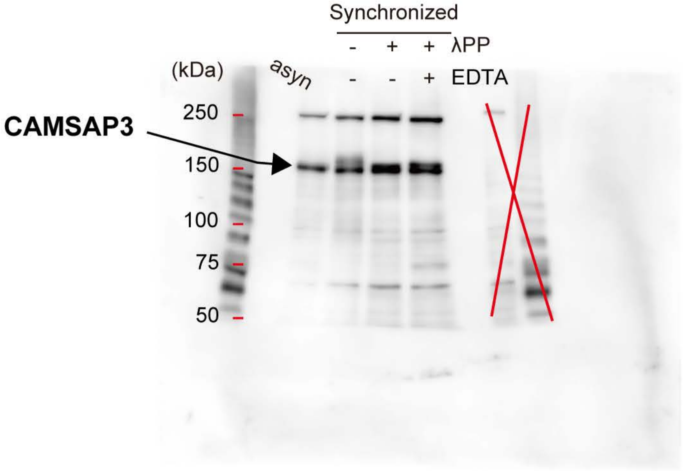

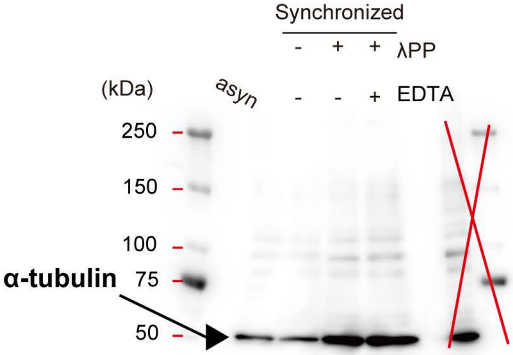

Fig S5A

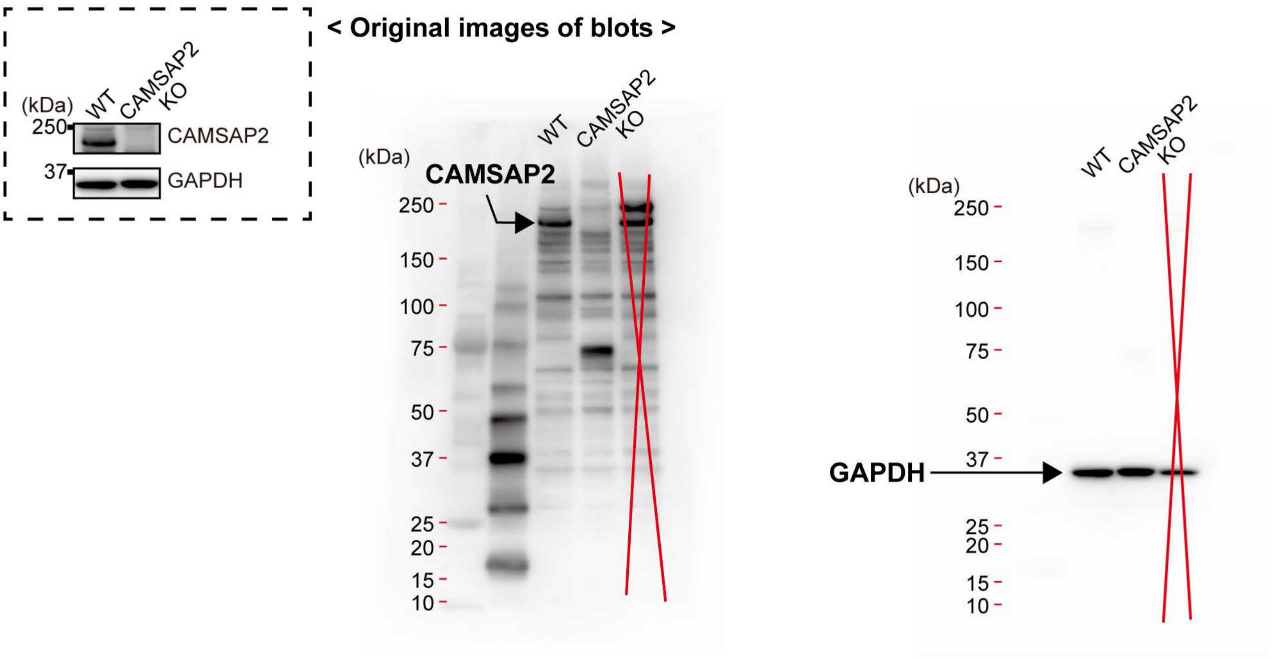

Fig S5B

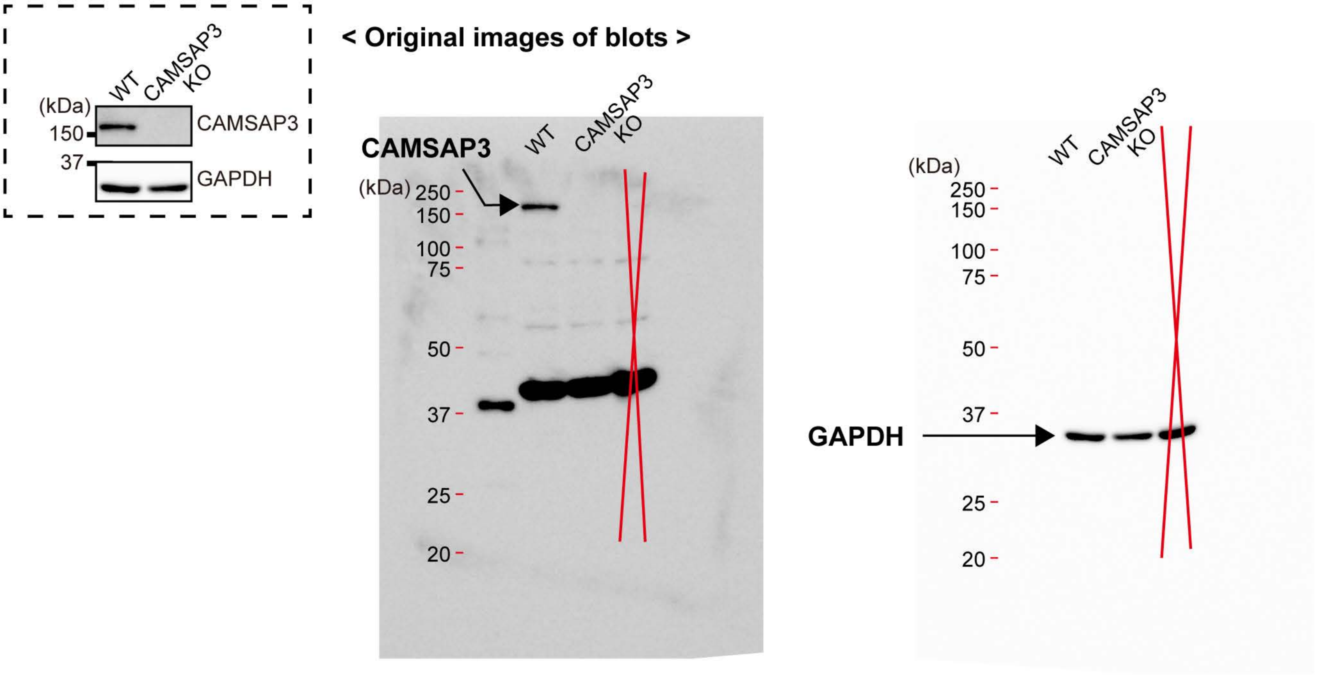

Fig S7B

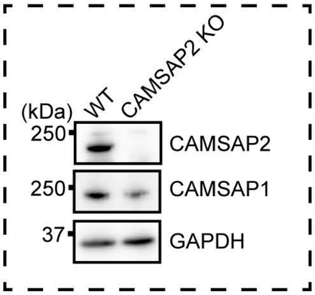

< Original images of blots >

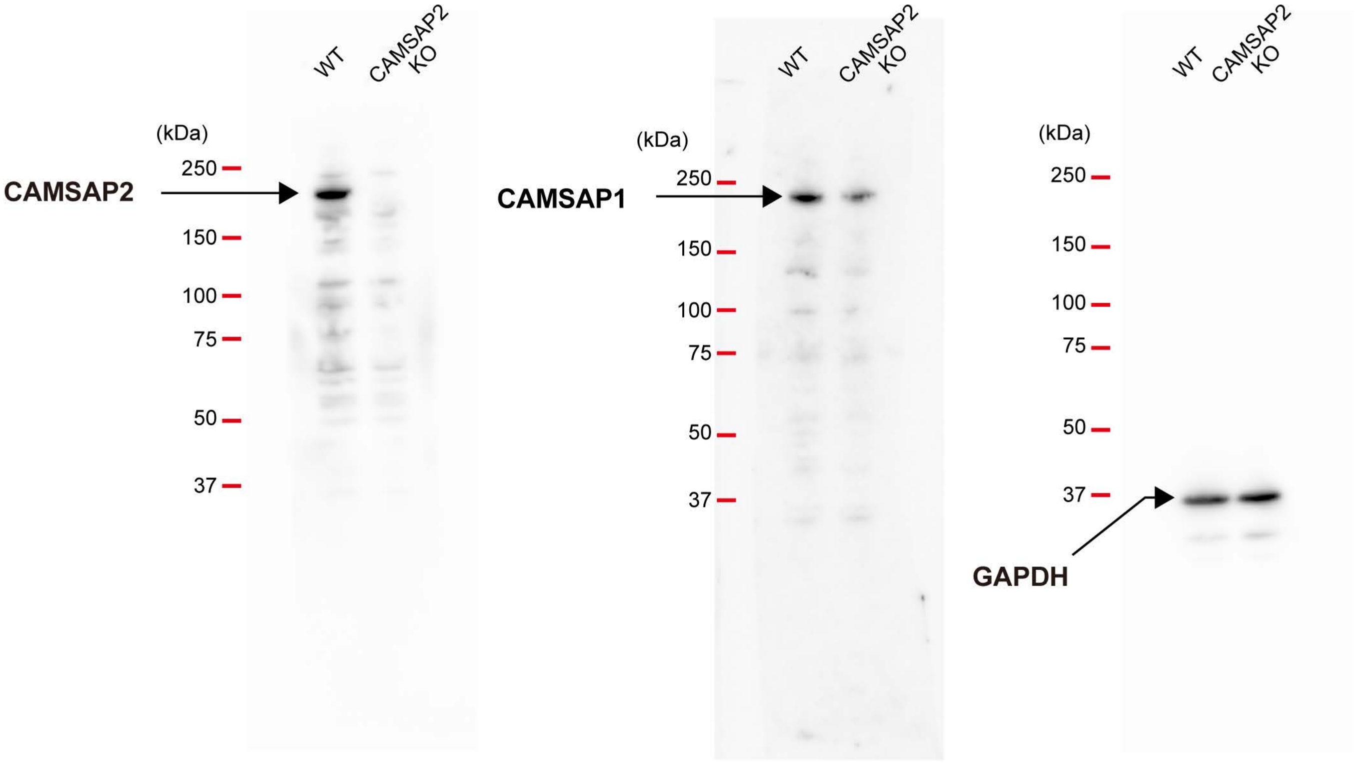

Fig S7E

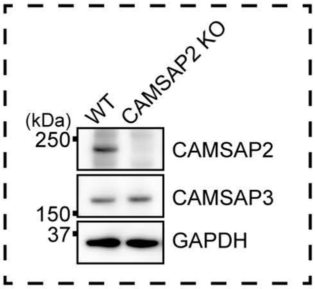

< Original images of blots >

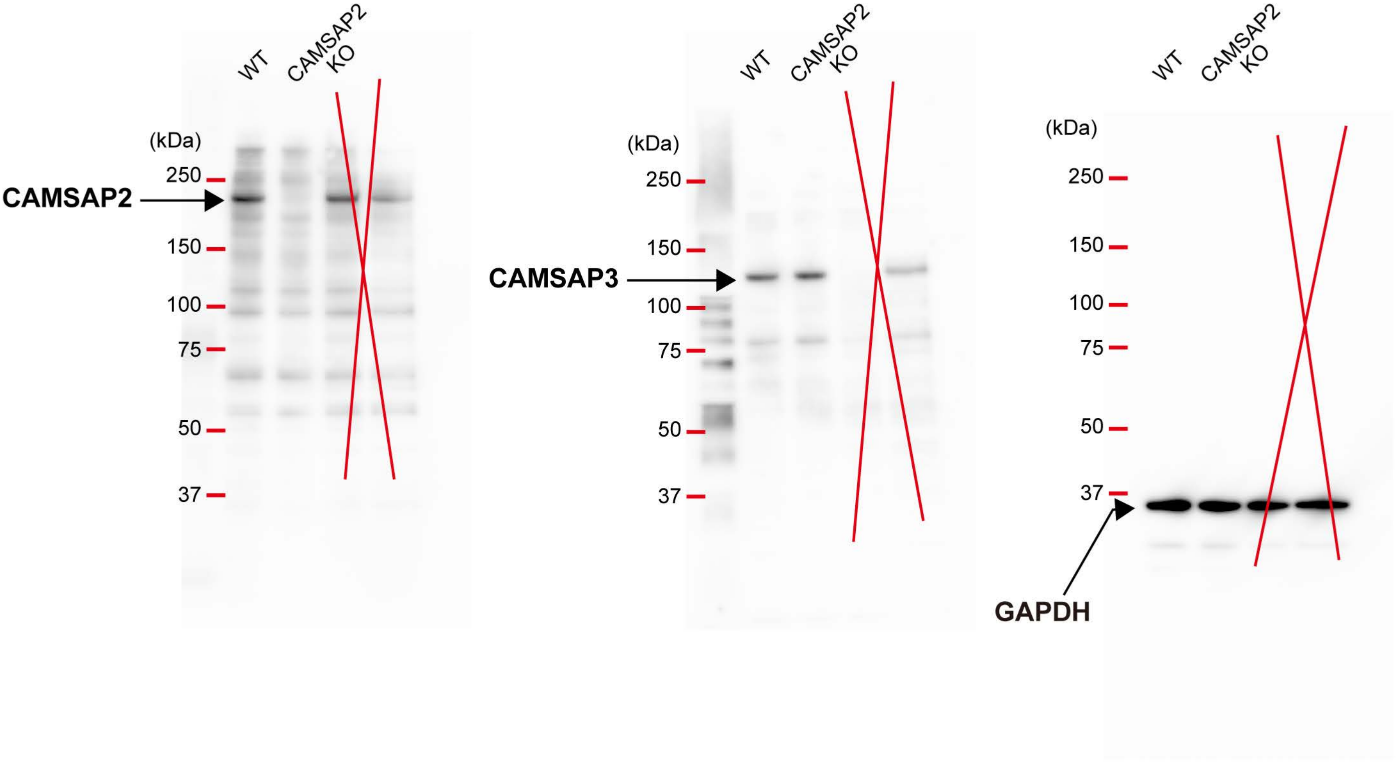

Fig S9

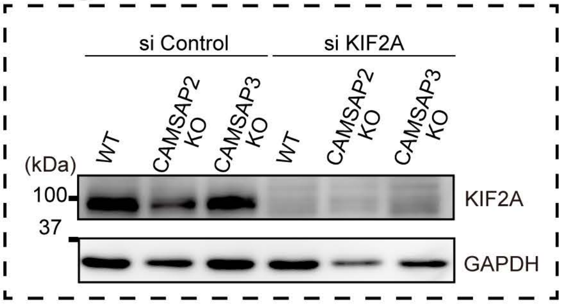

< Original images of blots >

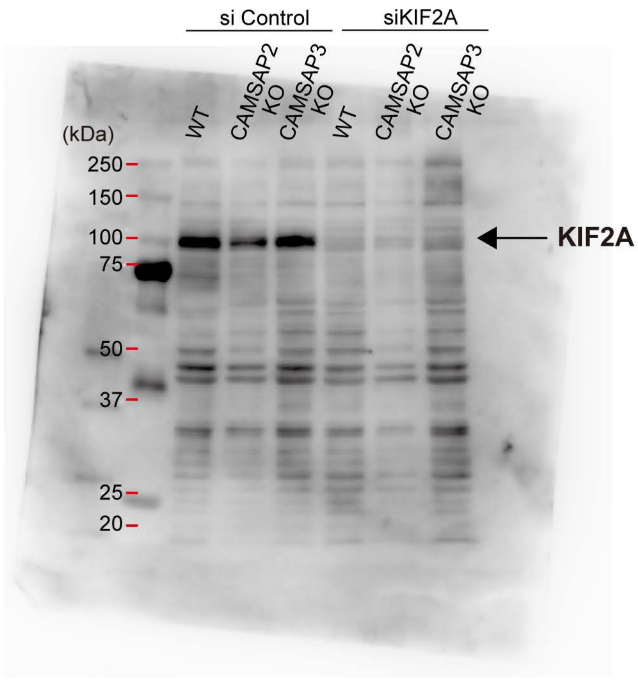

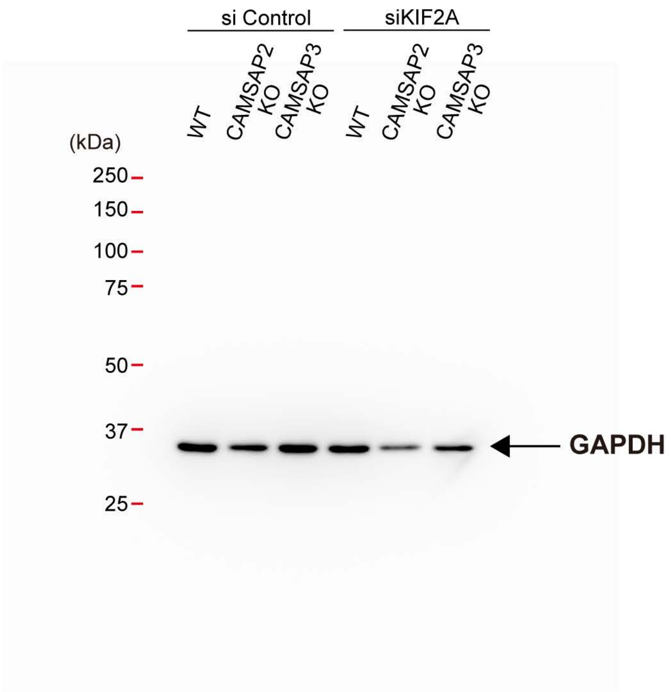

Fig S10A

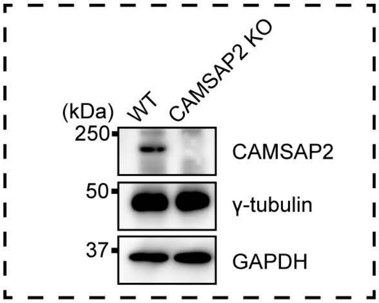

< Original images of blots >

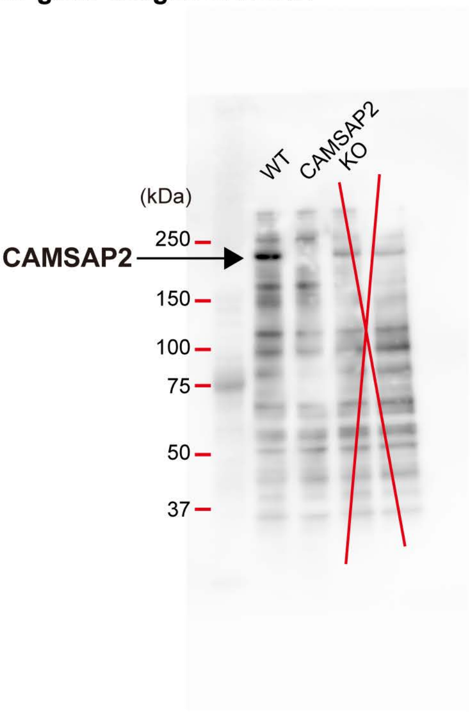

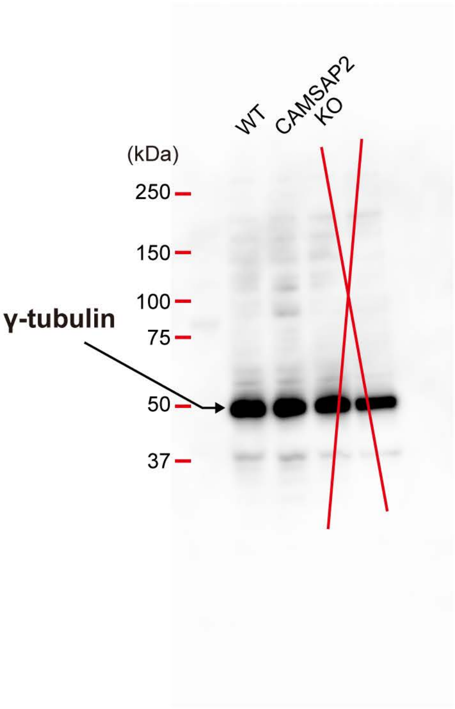

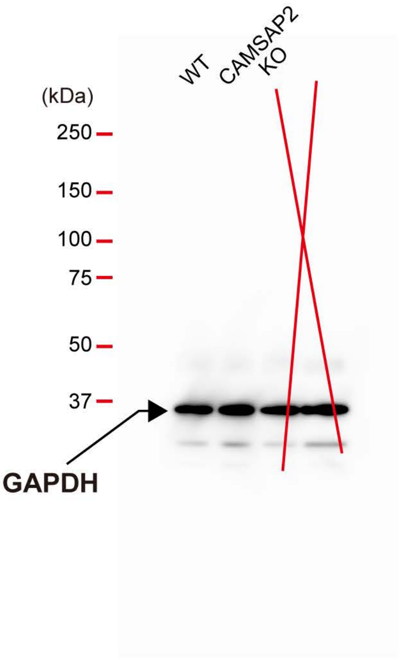

Fig S10B

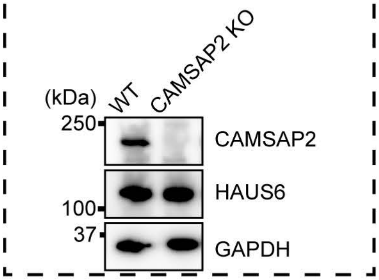

< Original images of blots >

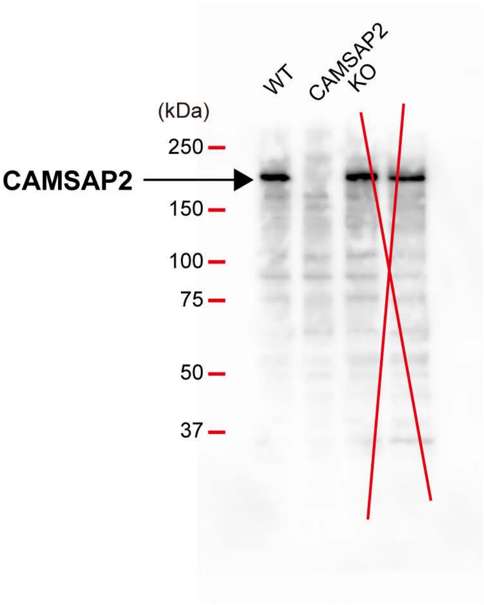

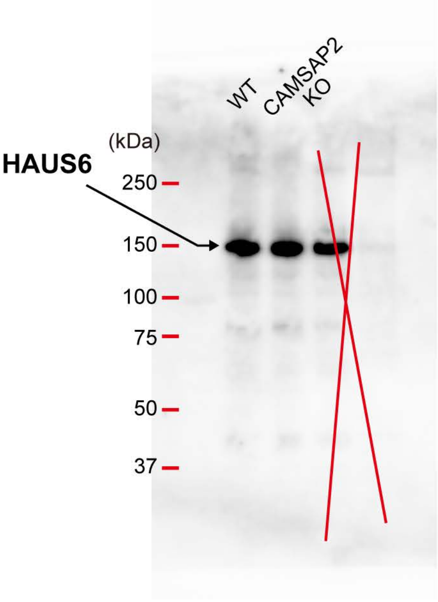

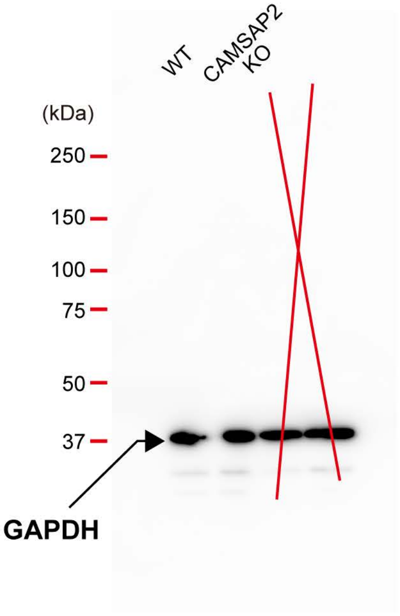

Fig S10C

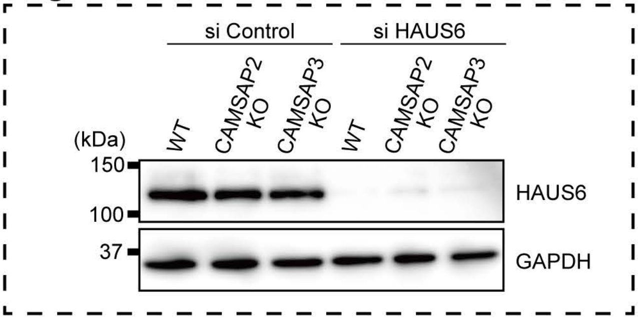

< Original images of blots >

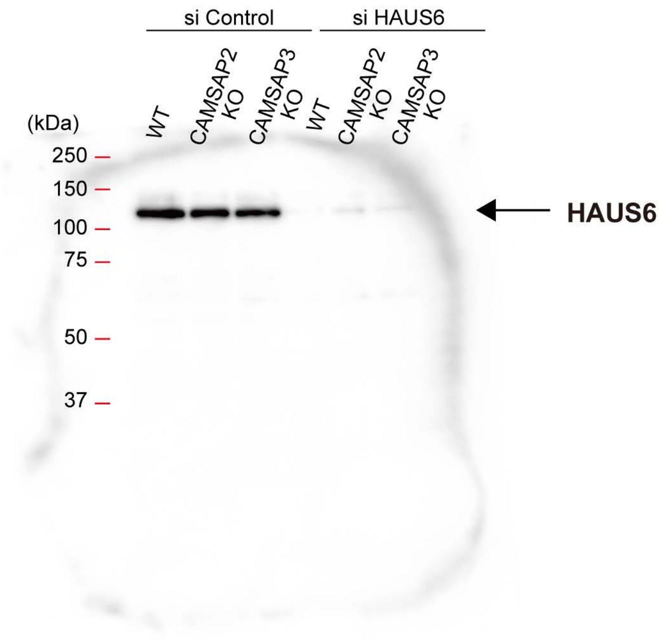

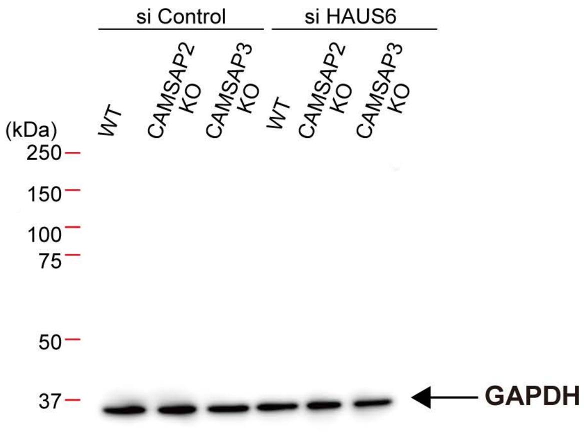

Supplement: S1 Raw images — (PDF) [file pone.0308150.s014.pdf]
