# Supplementary material for: CAMSAP2 is required for bridging fiber assembly to ensure mitotic spindle assembly and chromosome segregation in human epithelial Caco-2 cells
Source: PLoS One. 2025 Jan 9;20(1):e0308150. doi: 10.1371/journal.pone.0308150 (PMC11717264; doi:10.1371/journal.pone.0308150)
Supplement: S1 File — (DOCX) [file pone.0308150.s016.docx]

**Supplementary Information**

**CAMSAP2 is required for bridging fiber assembly to ensure mitotic spindle assembly and chromosome segregation in human epithelial Caco-2 cells**

Tables S1 and S2

Figures S1–S11

Reference for Supplementary Information

**Table S1. Primers used to construct EB1-mCherry2 cell lines**

(XLSX)

**Table S2. Kinetics of spindle elongation speed over time during anaphase**

(XLSX)

The data used for drawing Fig 5H. Spindle elongation speed was calculated every 20 seconds for 5 minutes in anaphase (μm/sec). Values are shown as averages of observed cells (n = 16 [WT] and 20 [CAMSAP2 KO] cells at each timepoint). asterisks = P < 0.05, Student's t-test.

**Fig S1. Endogenous localization of CAMSAP2 in interphase and mitosis**

Endogenous localization of CAMSAP2 in each cell cycle stage of Caco-2 WT cells (A) and CAMSAP2 KO cells (B). Cells were fixed with methanol and stained for α-tubulin (green), CAMSAP2 (red) and DAPI (blue). The boxed regions have been enlarged, brightness-adjusted, and shown in the inset. Arrowheads, punctum localization of CAMSAP2 at microtubule ends. CAMSAP2 punctae seen in interphase WT cells were undetectable in CAMSAP2 KO cells (B). CAMSAP2 signals at spindle poles seen in WT mitosis (A) were concluded as non-specific signals, as similar signals were also detected in CAMSAP2 KO cells (B). Scale bars; 10 μm.

**Fig S2. Endogenous localization of CAMSAP3 in interphase and mitosis**

Endogenous localization of CAMSAP3 in each cell cycle stage of Caco-2 WT cells (A) and CAMSAP3 KO cells (B). Cells were fixed with methanol and stained for α-tubulin (green), CAMSAP3 (red) and DAPI (blue). The boxed region has been enlarged, brightness-adjusted and shown in the inset. CAMSAP3 punctae (arrowheads) was observed in interphase WT cells (A) but was undetectable in CAMSAP3 KO cells (B). Scale bars; 10 μm.

**Fig S3. Localization of episomally expressed EGFP-tagged Camsap2 and Camsap3**

Localization of EGFP-Camsap2 (A) and EGFP-Camsap3 (B) overexpressed from plasmids in interphase and mitosis (metaphase). Representative images acquired using an LSM980 Airyscan are shown. Cells were fixed with methanol and stained for GFP (green), β-tubulin (red) and DAPI (blue). Single z-planes corresponding to the boxed regions have been enlarged, brightness-adjusted and shown in insets. Arrowheads indicate EGFP-Camsap2 (A) and EGFP-Camsap3 (B) punctae at microtubule ends. (A) EGFP-Camsap2 punctae were undetectable in metaphase. (B) EGFP-Camsap3 signals were detected at spindle poles in mitosis when overexpressed. Scale bar; 10 μm.

**Fig S4. Synchronization of cells by the STLC treatment**

WT cells were synchronized to metaphase with 5μM STLC. Mitotic index with or without the STLC treatment was analyzed as in Fig 1D. n = 213 DMSO-treated cells (control) and n = 238 STLC-treated cells in an experiment. Mitotic index was 4.23% in asynchronous DMSO-treated cells, which was increased to 36.55% in STLC-treated cells.

**Fig S5. Protein expression and cell cycle analyses in CAMSAP2 KO and CAMSAP3 KO cells**

Depletion of CAMSAP2 in CAMSAP2 KO cells (A) and depletion of CAMSAP3 in CAMSAP3 KO cells (B) were confirmed by western blotting. GAPDH, loading control. MW, kDa. (C) FACS analyses for WT, CAMSAP2 KO and CAMSAP3 KO cells. Cells were stained with propidium iodide and analyzed using the cell analyzer Cytomics FC500MPL. No apparent peak differences were observed among the observed cells. Three independent experiments were conducted, and the representative data are shown.

**Fig S6. Correlation assays between the spindle length and cell diameter**

Correlation between the spindle length and cell diameter was tested in WT and CAMSAP2 KO cells. The dataset used in Fig 2A were shared with the test. Mad2-negative spindles were exclusively chosen as metaphase cells for quantification. Neither WT nor CAMSAP2 KO show correlation between the spindle length and cell diameter. n = 79 (WT) and 86 (CAMSAP2 KO) cells from 4 independent experiments. R, correlation coefficient.

**Fig S7. CAMSAP2 KO phenotypes can be explained by the loss of CAMSAP2 per se**

(A) Rescue experiments for CASMAP2 KO cells with episomal plasmids containing the EGFP-Camsap2 construct. Mad2-negative spindles were exclusively chosen as metaphase ones, and the spindle length and the degree of displaced centrosomes were quantified. The spindle length (left) was measured based on β-tubulin signals. n = 50 (WT + EGFP), 49 (CAMSAP2 KO + EGFP), and 41 (CAMSAP2 KO + EGFP-Camsap2) cells from 3 independent experiments. ****P < 0.0001, n.s.: P > 0.05, Welch’s t-test. Degree of displaced centrosomes (right) was calculated based on γ-tubulin signals at spindle poles as in Fig 2H. n = 55 (WT + EGFP), 52 (CAMSAP2 KO + EGFP) and 66 (CAMSAP2 KO + EGFP-Camsap2) cells from 3 independent experiments. ****P < 0.0001, Welch’s t-test. n.s.: P > 0.05, Welch's t-test. Those indices for cells harboring the EGFP-Camsap2 plasmid were restored to the level comparable to WT + EGFP (control). (B) Western blotting for CAMSAP1. The band intensity of CAMSAP1 was normalized with the GAPDH (control) intensity. MW (kDa) is shown in the left. Mean ± SD from 3 independent experiments. n.s.: P > 0.05, Student's t-test. (C) Localization of 3×GFP-CAMSAP1 (red, three tandem copies of GFP are fused with CAMSAP1 at the N-terminus) to the metaphase spindle in WT and CAMSAP2 KO cells. Microtubules were stained with SiR-tubulin (green) and DNA with Hoechst (blue). CAMSAP1 signals were detected on spindle microtubules both in WT and CAMSAP2 KO cells. Scale bar; 10 μm. (D) Endogenous localization of CAMSAP1 to the spindle. Cells were fixed with methanol and stained for α-tubulin (green), CAMSAP1 (red) and DAPI (blue). CAMSAP1 signals were detected at spindle poles and microtubules in WT and CAMSAP2 KO cells. The background intensity was subtracted to calculate the CAMSAP1 intensity on the spindle. No significant differences in the amount of CAMSAP1 were seen between WT and CAMSAP2 KO cells. n = 81 (WT) and 61 (CAMSAP2 KO) cells, 3 independent experiments. n.s.: P > 0.05, Student's t-test. Scale bars; 10 μm. (E) Western blotting for CAMSAP3 in CAMSAP 2KO cells. The band intensity of CAMSAP3 was normalized with the GAPDH (control) intensity. MW (kDa) is shown in the left. Mean ± SD from 3 independent experiments. n.s.: P > 0.05, Student's t-test.

**Fig S8. CAMSAP2 localization in interphase and mitotic CAMSAP3 KO cells**

Endogenous localization of CAMSAP2 in interphase and each mitotic phase in CAMSAP3 KO cells. Methanol-fixed cells were stained for α-tubulin (green), CAMSAP2 (red) and DAPI (blue). The boxed region has been enlarged, brightness-adjusted and shown in the inset. Arrowheads, stretched CAMSAP2 signals at the microtubule ends, as previously reported [1]. CAMSAP2 signals were undetectable in mitotic cells. CAMSAP2 signals at spindle poles were concluded as non-specific ones as were also detected in CAMSAP2 KO cells (see Fig S1B). Scale bars; 10 μm.

**Fig S9. Depletion of KIF2A by siRNA**

Depletion of KIF2A was confirmed by western blotting in WT, CAMSAP2 KO and CAMSAP3 KO cells with (si KIF2A) or without (si Control) KIF2A knockdown. GAPDH, the loading control. MW (kDa) is shown in the left.

**Fig S10. Mitotic phenotypes caused by co-depletion of CAMSAP2 and HAUS6**

(A,B) Western blotting for γ-tubulin (A) and HAUS6 (B) in WT and CAMSAP2 KO cells. The band intensities were normalized with the GAPDH (control) intensity. MW (kDa) is shown in the left. Mean ± SD from 3 independent experiments. n.s.: P > 0.05, Student's t-test. (C) Depletion of HAUS6 by siRNA was confirmed by western blotting. GAPDH, loading control. (D) The spindle length in HAUS6/control-depletion in WT and CAMSAP2 KO cells. Cells were fixed with 2% PFA and stained for α-tubulin (green), BubR1 (red) and DNA (blue). Representative images acquired by an LSM980 Airyscan are shown. BubR1-negative spindles were exclusively chosen as metaphase ones to measure the metaphase spindle length based on α-tubulin signals. n = 55 cells (si control WT), 58 (si HAUS6 WT), 47 (si control CAMSAP2 KO) and 66 (si HAUS6 CAMSAP2 KO) from 3 independent experiments. Boxplots (left) indicate 25th percentile, median and 75th percentile. ****P < 0.0001, **P < 0.01 Student's t-test. The percentage decrease of spindle length (right) in WT and CAMSAP2 KO cells upon HASU6 co-depletion normalized by control cells. HAUS6 depletion reduced the spindle size to a similar extent in WT and in CAMSAP2 KO cells, although the effect appeared slightly more in the CAMSAP2 KO despite of no statistical significance. This indicates that the effect of CAMSAP2 in spindle length determination is largely through the Augmin pathway. Mean ± SEM of 3 independent experiments. n.s.: P > 0.05, Student’s t-test. Scale bar; 10 μm. (E) The γ-tubulin intensity in the spindle excluding spindle poles. Cells were fixed with 2% PFA and stained for α-tubulin (green), γ-tubulin (red) and DAPI (blue). The background intensity off the spindle was subtracted to calculate the γ-tubulin intensity on the spindle. The significant reduction in the amount of γ-tubulin intensity in si control WT and si control CAMSAP2 KO was no longer seen si HAUS6 WT and si HAUS6 CAMSAP2 KO. n = 51 cells (si control WT), 44 (si HAUS6 WT), 50 ( si control CAMSAP2 KO), and 47 (si HAUS6 CAMSAP2 KO) from 3 independent experiments. Boxplots indicate 25th percentile, median and 75th percentile values. ****P < 0.0001, Welch’s t-test, n.s. > 0.05, Student’s t-test. Scale bar; 10 μm. (F) Organization of bridging fibers in single and double depletion. Representative images of a single z-plane acquired by an LSM980 Airyscan are shown. WT and CAMSAP2 KO cells with (si HAUS6) or without (si Control) HAUS6 knockdown were fixed with 2% PFA and stained for α-tubulin (green), CREST (red) and DAPI (blue). Boxed regions including representative sister kinetochore fibers have been magnified, brightness-adjusted and shown in insets. Arrowheads indicate a bridging fiber. Schematics are shown below each image. Frequencies (%) of sister kinetochore fibers that accompany bridging fibers behind are shown (bottom). The significant reduction was seen in bridging fibers in si control WT and si control CAMSAP2 KO, which was no longer seen in si HAUS6 WT and si HAUS6 CAMSAP2 KO. n = 28 pairs of kinetochores from 14 cells (si control WT), 38 from 20 cells (si control CAMSAP2 KO), 35 from 14 cells (si HAUS6 WT), and 40 from 18 cells (si HAUS6 CAMSAP2 KO) from 3 independent experiments. Mean ± SD of 3 independent experiments. *P < 0.05, Welch's t-test. n.s.: P > 0.05, Student's t-test. Scale bar; 10 μm.

**Fig S11 A hypothetic model for spindle assembly in WT and CAMSAP KO cells.**

(A) Schematics for hypotheses: how CAMSAP2 contributes to spindle assembly upon entry into mitosis. See text for details. (B) Simultaneous observation of CAMSAP2 and CAMSAP3 localization in Caco-2 cells. Either CAMSAP2-GFP (top) or CAMSAP3-GFP (bottom) was ectopically expressed in wild-type cells. Cells were fixed with methanol and stained with anti-GFP (green), α-tubulin (gray), and DAPI (blue). CAMSAP3 (top) or CAMSAP2 (bottom) was stained with anti-CAMSAP3 or anti-CAMSAP2 antibody, respectively. Boxed regions are enlarged to visualize CAMSAP2 and CAMSAP3 punctae at microtubule ends. Microtubule ends with CAMSAP2 only (yellow arrowheads) or CAMSAP3 only (blue arrowheads) are indicated. Scale bars; 10 μm. The chart shows percentages of microtubules decorated with either or both CAMSAP2 and CAMSAP3 in interphase (N = 3 cells, n = 102, 97, 118 microtubules for +pEGFP-CAMSAP2; n = 74, 118, 78 for +pEGFP-CAMSAP3. Data are presented as mean ± standard error of the mean (SEM). ****P < 0.0001, ***P < 0.001, **P < 0.01, *P < 0.05, One-way ANOVA followed by Tukey’s multiple comparison tests.

**Reference for Supplementary Information**

1. Tanaka N, Meng W, Nagae S, Takeichi M. Nezha/CAMSAP3 and CAMSAP2 cooperate in epithelial-specific organization of noncentrosomal microtubules. Proc National Acad Sci. 2012;109: 20029–20034. doi:10.1073/pnas.1218017109
